# A morphospace of functional configuration to assess configural breadth based on brain functional networks

**DOI:** 10.1162/netn_a_00193

**Published:** 2021-08-30

**Authors:** Duy Duong-Tran, Kausar Abbas, Enrico Amico, Bernat Corominas-Murtra, Mario Dzemidzic, David Kareken, Mario Ventresca, Joaquín Goñi

**Affiliations:** School of Industrial Engineering, Purdue University, West Lafayette, IN, USA; Purdue Institute for Integrative Neuroscience, Purdue University, West Lafayette, IN, USA; School of Industrial Engineering, Purdue University, West Lafayette, IN, USA; Purdue Institute for Integrative Neuroscience, Purdue University, West Lafayette, IN, USA; School of Industrial Engineering, Purdue University, West Lafayette, IN, USA; Purdue Institute for Integrative Neuroscience, Purdue University, West Lafayette, IN, USA; Institute of Bioengineering/Center for Neuroprosthetics, Ecole Polytechnique Fédérale de Lausanne, Lausanne, Switzerland; Department of Radiology and Medical Informatics, University of Geneva, Switzerland; Department of Zoology, Institute of Biology, Karl-Franzens University Graz, Graz, Austria; Department of Neurology, Indiana University School of Medicine, Indianapolis, IN, USA; Department of Neurology, Indiana University School of Medicine, Indianapolis, IN, USA; School of Industrial Engineering, Purdue University, West Lafayette, IN, USA; Purdue Institute of Inflammation, Immunology, and Infectious Disease, Purdue University, West Lafayette, IN, USA; School of Industrial Engineering, Purdue University, West Lafayette, IN, USA; Purdue Institute for Integrative Neuroscience, Purdue University, West Lafayette, IN, USA; Weldon School of Biomedical Engineering, Purdue University, West Lafayette, IN, USA

**Keywords:** Functional reconfiguration, Functional configural breadth, Resting-state networks, Functional connectomes

## Abstract

The quantification of human brain functional (re)configurations across varying cognitive demands remains an unresolved topic. We propose that such functional configurations may be categorized into three different types: (a) network configural breadth, (b) task-to task transitional reconfiguration, and (c) within-task reconfiguration. Such functional reconfigurations are rather subtle at the whole-brain level. Hence, we propose a mesoscopic framework focused on functional networks (FNs) or communities to quantify functional (re)configurations. To do so, we introduce a 2D network morphospace that relies on two novel mesoscopic metrics, trapping efficiency (TE) and exit entropy (EE), which capture topology and integration of information within and between a reference set of FNs. We use this framework to quantify the network configural breadth across different tasks. We show that the metrics defining this morphospace can differentiate FNs, cognitive tasks, and subjects. We also show that network configural breadth significantly predicts behavioral measures, such as episodic memory, verbal episodic memory, fluid intelligence, and general intelligence. In essence, we put forth a framework to explore the cognitive space in a comprehensive manner, for each individual separately, and at different levels of granularity. This tool that can also quantify the FN reconfigurations that result from the brain switching between mental states.

## Author Summary

Understanding and measuring the ways in which human brain connectivity changes to accommodate a broad range of cognitive and behavioral goals is an important undertaking. We put forth a *mesoscopic* framework that captures such changes by tracking the topology and integration of information within and between functional networks (FNs) of the brain. Canonically, when FNs are characterized, they are separated from the rest of the brain network. The two metrics proposed in this work, trapping efficiency and exit entropy, quantify the topological and information integration characteristics of FNs while they are still embedded in the overall brain network. Trapping efficiency measures the module’s ability to preserve an incoming signal from escaping its local topology, relative to its total exiting weights. Exit entropy measures the module’s communication preferences with other modules/networks using information theory. When these two metrics are plotted in a 2D graph as a function of different brain states (i.e., cognitive/behavioral tasks), the resulting morphospace characterizes the extent of network reconfiguration between tasks (functional reconfiguration), and the change when moving from rest to an externally engaged “task-positive” state (functional preconfiguration), to collectively define network configural breadth. We also show that these metrics are sensitive to subject, task, and functional network identities. Overall, this method is a promising approach to quantify how human brains adapt to a range of tasks, and potentially to help improve precision clinical neuroscience.

## INTRODUCTION

Human behavior arises out of a complex interplay of functional dynamics between different brain networks (Bassett & Gazzaniga, 2011). These interactions are reflected in functional network (FN) reconfigurations as subjects perform different tasks or are at rest (Amico, Abbas, et al., 2019; Amico et al., 2020; Cole, Bassett, Power, Braver, & Petersen, 2014). One of the network neuroscience challenges is to develop a comprehensive framework to quantify the brain network (re)configurations across different mental states and cognitive tasks. To that end, configurations across a collection of cognitive tasks can be conceptualized at three distinct levels of granularity:

■  **Network configural breadth** represents, for an FN, a given individual’s repertoire of cognitive and emotional states through functional configurations while performing different tasks. In practice, how well the entire “cognitive space” (Varona & Rabinovich, 2016; Varoquaux et al., 2018) is sampled depends on the number and choice of the tasks. This concept is inspired by Schultz and Cole (2016).
■  **Task-to-task transitional reconfiguration** represents the specific shift in network functional configuration when a subject switches between cognitive/mental tasks (Douw, Wakeman, Tanaka, Liu, & Stufflebeam, 2016; Gonzalez-Castillo et al., 2015). For instance, task transitions and accompanying reconfigurations will occur when a subject transitions from quiet reflection to engage in a spatial problem-solving task, or from a lexical retrieval to a decision-making paradigm.
■  **Within-task reconfiguration** represents specific network functional configuration changes that may occur within a single task. This phenomenon has been assessed at the whole-brain level, showing the presence of distinct brain states within a task (Bassett et al., 2011; Betzel, Satterthwaite, Gold, & Bassett, 2017; J. M. Shine et al., 2016; J. M. Shine et al., 2019; J. M. Shine & Poldrack, 2018).

While brain network configural properties are task and subject dependent (Schultz & Cole, 2016), task-induced functional (re)configurations are rather subtle in whole-brain functional connectomes, even when comparing task with rest (Cole et al., 2014). In addition, mesoscopic structures (e.g., functional networks of the brain) exhibit modular characteristics that adapt to cognitive demands without significantly affecting the rest of the system where higher levels of cognition emerge through the changing interactions of subsystems, instead of pairwise edge-level interactions (Bassett et al., 2011). Hence, a mesoscopic scale (as the one provided by functional networks or communities/modules) may uncover differential patterns of (re)configuration (Mohr et al., 2016), across functional subcircuits, which might otherwise not be detectable at other scales. Traditionally, a mesoscopic assessment of functional brain networks would involve the *detection* of functional communities (Sporns & Betzel, 2016) either based on topology (density-based; Newman, 2006a, 2006b) or based on the information flow (flow-based; Rosvall, Axelsson, & Bergstrom, 2009; Rosvall & Bergstrom, 2008). These approaches, however, are not designed to *track* the dynamic behavior of a priori set of communities across time, tasks, and/or subjects. The primary aim of this work is to clearly define and quantify different configurations that FNs can assume, as well as measure their nature of reconfigurations switching between a seemingly infinite number of cognitive states. From a graph-theoretical perspective, FNs and their corresponding reconfigurations are described by two attributes: topology and communication. From a system dynamic perspective, FNs can be characterized by segregation and integration (Sporns, 2013) properties across which the human brain reconfigures across varied cognitive demands (J. Shine et al., 2018; J. M. Shine et al., 2016; J. M. Shine et al., 2019; J. M. Shine & Poldrack, 2018). To formally capture these diverse characteristics of FNs, we constructed a mathematically well-defined and well-behaved 2D “mesoscopic morphospace” based on two novel measures defined for nonnegative, undirected, weighted functional connectomes: trapping efficiency (**TE**) and exit entropy (**EE**). Trapping efficiency captures the level of segregation/integration of a functional network embedded in the rest of the functional connectome and quantifies the extent to which a particular FN “traps” an incoming signal. Exit entropy captures the specificity of integration of an FN with the rest of the functional connectome, and quantifies the uncertainty as to where (in terms of exit nodes) that same signal would exit the FN. In summary, this mesoscopic morphospace is a representation of the cognitive space as explored within and between cognitive states, as reflected by brain activity in fMRI. Such representation relies on FN reconfigurations that can be tracked, at an individual level, and at different granularity levels in network (re)configurations.

**Figure 1.**
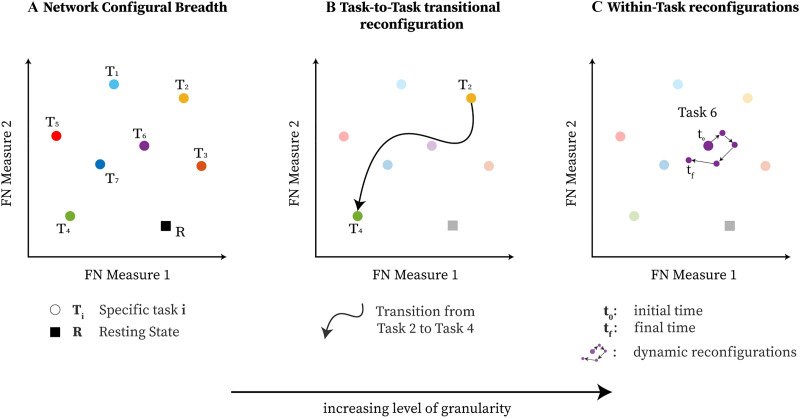
The three types of brain (re)configurations that can be represented by a mathematical space parameterized by, in this case, two generic phenotypic measures of functional communities of the brain: (**A**) network configural breadth, which represents changes across a number of cognitive demands; (**B**) task-to-task transitional reconfiguration; and (**C**) within-task reconfiguration.

By using this 2D **TE**, **EE**-based morphospace, we formally study network configural breadth (Figure 1A), the most global and coarse grain exploration of the cognitive space, and its subsequent functional configuration components. To that end, we formally define measures of (a) functional reconfiguration (capacity of an individual to reconfigure across widely differing cognitive operations) and (b) functional preconfiguration (efficiency of transition from resting state to task-positive state (Schultz & Cole, 2016)), for potentially any community or FN. These measures are quantified for resting-state networks (Yeo et al., 2011) on the 100 unrelated subjects from the Human Connectome Project (HCP) dataset. We then study how such quantification is related to measures of cognitive abilities, such as fluid intelligence.

## A MESOSCOPIC MORPHOSPACE OF FUNCTIONAL CONFIGURATIONS

The *mesoscopic morphospace* proposed here is a two-dimensional space built upon trapping efficiency and exit entropy measures for assessing functional networks or communities of functional connectomes. In this framework, functional connectomes must be undirected (symmetrical) weighted graphs, with *nonnegative* functional couplings. This framework allows for any a priori partition into functional communities. In this work, we assess the resting-state functional networks as proposed by Yeo et al. (2011) as the a priori FNs. Also, we use functional connectivity (without incorporating structural connectivity information), which is a quantification of statistical dependencies between BOLD time series of brain regions, and it can be used as a proxy of communication dynamics in the brain (Fornito, Zalesky, & Bullmore, 2016). Under this section, further technical details that are not mentioned in the main text will be directed to different subsections in the Supporting Information.

### Computing Mechanistic Components for Morphospace Measures

A mesoscopic morphospace is constructed to assess functional network behaviors through two focal lenses: level of segregation/integration (using graph topology), and specificity of integration (using information theory). We first define all necessary components to compute **TE** and **EE** as follows:

(a)  The whole-brain functional connectome (FC) is graph-theoretically denoted by *G*(*V*, *E*), where *V* is the set of vertices (represented by the regions of interest, ROIs) and *E* is the set of edges (quantified by functional couplings between pairs of ROIs). The whole-brain FC is mathematically represented by an adjacency structure denoted as **A** = [*w*_*ij*_], where *i*, *j* are indexed over vertex set *V* and *w*_*ij*_ ∈ [0, 1] are functional couplings.
(b)  Using a predefined set of FNs, a functional community (graph-theoretically denoted as *G*_𝒞_(*V*_𝒞_, *E*_𝒞_) or 𝒞 for short) is defined to have the corresponding node set *V*_𝒞_ ⊂ *V* and edge set *E*_𝒞_ ⊂ *E* for which the union over all FNs exhaust the vertex and edge set of *G* such that
  $$\cup V_{𝒞}=V\;\text{and}\;\cup E_{𝒞}=E.$$
(c)  For a given functional community 𝒞 ⊂ *G*, define the set of states (or equivalently, vertices) *S* that contains the set of transient states (denoted as *S*_*trans*_ = *V*_𝒞_), and absorbing states (denoted as *S*_*abs*_ = {*j* | *w*_*ij*_ > 0; *j* ∉ *V*_𝒞_, ∀ *i* ∈ *V*_𝒞_}) such that
  $$S=S_{\text{trans}}\cup S_{abs}.$$
(d)  We mathematically denote a whole-brain FC as **A** = [*w*_*ij*_] (see the Constructing Functional Connectomes section of the Supporting Information for more details), where *i* and *j* are brain regions (from now on denoted as vertices or states) of the specified parcellation or atlas. Each matrix **A** represents a single subject, single session, single task whole-brain FC. We assess the whole-brain FC with respect to organizations into FNs, here denoted by 𝒞. For a specific **A** and a specific 𝒞, we obtain an induced submatrix **A**_𝒞_ by extracting the corresponding rows and columns of matrix **A** using only the vertices that belong to *S*, which results in the following matrix:
  $$A_{𝒞}\in {\left(0,1\right)}^{\left|S\right|\times \left|S\right|}.$$
  We note that the row and column order of the states (or vertices) of **A**_𝒞_ respects the order of *S* = *S*_*trans*_ ∪ *S*_*abs*_ with transient states followed by absorbing ones, which results in a blockage structure:
  $$\begin{array}{c} \;\text{Transient}\;\text{Absorbing}\\ A_{𝒞}=\begin{array}{c} \text{Transient}\\ \text{Absorbing} \end{array}\left(\begin{array}{cc} A\left(S_{\text{trans}},S_{\text{trans}}\right) & A\left(S_{\text{trans}},S_{abs}\right)\\ A\left(S_{abs},S_{\text{trans}}\right) & A\left(S_{abs},S_{abs}\right) \end{array}\right), \end{array}$$
  where **A**(*S*_*trans*_, *S*_*trans*_) means that we extract the submatrix of **A** that corresponds to states in *S*_*trans*_ for the rows (first argument) and *S*_*trans*_ for the columns (second argument).
(e)  For any functional network 𝒞, using the induced adjacency structure **A**_𝒞_ in the previous step, define each vertex in *S* to be a state in the stochastic process and construct the corresponding terminating Markov chain by computing the following:
  ■  the normalization of **A**_𝒞_ by the nodal connectivity strength:
    $$\mathbb{Q}=D_{𝒞}^{-1}A_{𝒞}\in {\left(0,1\right)}^{\left|S\right|\times \left|S\right|},$$
    where **D**_𝒞_ is the weighted degree sequence matrix filled with the node strength (defined by the row [or equivalently, column] sum of **A**_𝒞_) in the diagonal entries and zeros for the off-diagonal elements:
    $$D_{𝒞}=\left[d_{ij}\right]=\left\{\begin{array}{c} \sum_{j=1}^{j=\left|V_{𝒞}\right|}w_{ij},\forall i=j\\ 0,\forall i\neq j \end{array}\right.,$$
    where *i*, *j* are indexed over *S*. Note that the order of rows and columns of ℚ and **D**_𝒞_ also respect the order of *S*.
  ■  the transition probability matrix of the terminating Markov chain:
    $$\begin{array}{c} \;\text{Transient}\;\text{Absorbing}\\ P=\begin{array}{c} \text{Transient}\\ \text{Absorbing} \end{array}\left(\begin{array}{cc} \mathbb{Q}\left(S_{\text{trans}},S_{\text{trans}}\right) & \mathbb{Q}\left(S_{\text{trans}},S_{abs}\right)\\ 0_{\left|S_{abs}\right|\times \left|S_{\text{trans}}\right|} & I_{\left|S_{abs}\right|} \end{array}\right), \end{array}$$
    where **0**_|*S*_*abs*_|×|*S*_*trans*_|_ is the matrix of all zeros (size |*S*_*abs*_| rows by |*S*_*trans*_| columns); **I**_|*S*_*abs*_|_ is identity matrix of size |*S*_*abs*_|; the index 𝒞 for ℚ and **P** is dropped for simplicity.
(f)  Using matrix **P**, we extract the submatrix induced by states in *S*_*trans*_ (denoted by **P**|_*S*_*trans*__). Note that **P**|_*S*_*trans*__ = ℚ(*S*_*trans*_, *S*_*trans*_) because rows and columns of **P** respect the order of *S*. We then compute the fundamental matrix (denoted as **Z**; Kemeny & Snell, 1960), which contains the mean number of steps a specific transient state in *S*_*trans*_ is visited, for any pair of transient states in *S*_*trans*_, before the random walker is absorbed by one of the states in *S*_*abs*_:
  $$Z={\left(I_{|S_{\text{trans}}}-P{|}_{S_{\text{trans}}}\right)}^{-1}\in {\mathbb{R}}_{+}^{\left|S_{\text{trans}}\right|\times \left|S_{\text{trans}}\right|}.$$
(g)  Compute the mean time to absorption (denoted as *τ*), which contains the mean number of steps that the random particle needs to be absorbed by one of the states in *S*_*abs*_, given that it starts in some state in *S*_*trans*_:
  $$\tau =Z1_{\left|S_{\text{trans}}\right|}\in {\mathbb{R}}_{+}^{\left|S_{\text{trans}}\right|\times 1},$$
  where **1**_|*S*_*trans*_|_ is the all one vector of size |*S*_*trans*_|.
(h)  Compute the absorption probability matrix (denoted as Ψ), which contains the likelihood of being absorbed by one of the absorbing states, given that the stochastic process starts in some transient state:
  $$\Psi =Z\left[P{|}_{S_{\text{trans}},S_{abs}}\right]\in {\mathbb{R}}_{+}^{\left|S_{\text{trans}}\right|\times \left|S_{abs}\right|},$$
  where **P**|_*S*_*trans*_,*S*_*abs*__ is the subtransition probability matrix induced from (row) state *S*_*trans*_ and (column) state *S*_*abs*_. Hence, **P**|_*S*_*trans*_,*S*_*abs*__ = ℚ(*S*_*trans*_, *S*_*abs*_).

### Module Trapping Efficiency

Module trapping efficiency, denoted as **TE** (unit: $\frac{\text{steps}}{\text{weight}}$), quantifies a module’s capacity to contain a random particle from leaving its local topology, that is, 𝒞. Specifically, through FN topology, we want to assess its level of *segregation*/*integration*, measured by the *L*_2_ norm of *τ* (unit: *steps*), that is, the mean time to absorption of nodes in 𝒞, normalized by its total exiting strength (unit: *weight*), measured by

$${𝓛}_{𝒞}=\sum_{i\in S_{\text{trans}},j\in S_{abs}}A_{ij}=A\left(S_{\text{trans}},S_{abs}\right).$$

Mathematically, trapping efficiency is quantified as follows:

$$TE=\frac{{\left\|\tau \right\|}_{2}}{{𝓛}_{𝒞}}.$$ (1)

**Figure 2.**
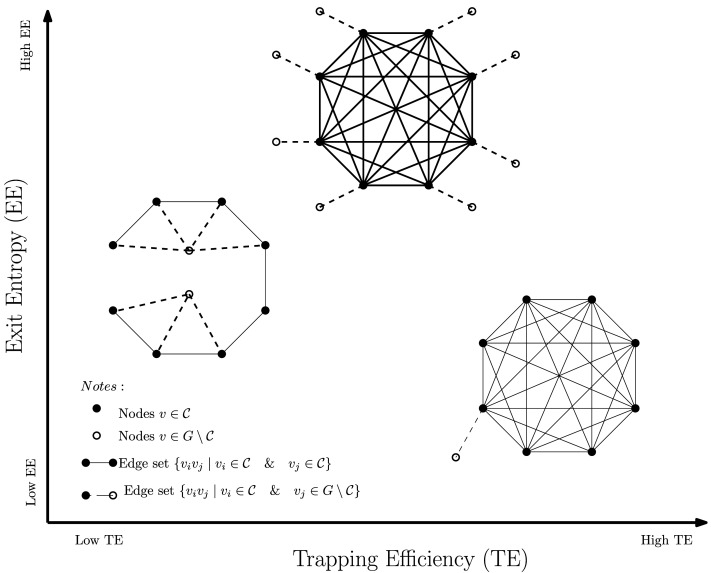
Morphospace measurements, examples. All three induced subgraphs have the same cardinality (|𝒞| = 8) with a different number of exits (connections to *G* \ 𝒞). Nonetheless, depending on their topological structures, the corresponding morphospace measurements (**TE** and **EE**) have rather distinct values.

We see that the mean time to absorption vector, *τ*, is dependent on both **density-based** (Fortunato, 2010; Newman, 2006b) and **flow-based** (Malliaros & Vazirgiannis, 2013; Rosvall et al., 2009; Rosvall & Bergstrom, 2008) modularity. The mean-time-to-absorption vector *τ* for which *τ*_*i*_ contains the average number of steps a random walker needs to escape the FN topology, given that it starts from node *i*. This means that the numerical values in *τ* are always greater than or equal to 1. We chose to use *L*_2_ norms because it squares the input values of the vector and thus enhances our capacity to quantify FN (re)configuration. On the other hand, the denominator 𝓛_𝒞_ is a simple statistical summary of the module “leakages” to the rest of the cortex. Since all the values in 𝓛_𝒞_ are between (0, 1), *L*_2_ norm would have diminished the differences across FNs. Hence, we chose *L*_1_ norm for the denominator. The role of 𝓛_𝒞_ is to account for potential differences in trapping efficiency due to community size. Numerically, higher **TE** indicates that a module is more segregated (or equivalently, less integrated). This is because the FN topology traps the incoming signal efficiently, relative to its exiting edges when embedded in the cortex. **TE** value ranges are given in Figure 2.

### Module Exit Entropy

Module exit entropy (denoted as **EE**, and in the range **EE** ∈ (0, 1] and unitless) assesses the normalized level of uncertainty in selecting an exiting node in *S*_*abs*_ of a random particle that starts in 𝒞 The exit entropy, denoted as 𝓗_*e*_, measures the level of uncertainty exiting node *j* ∈ *S*_*abs*_ (outside of the module) is preferred. Module exit entropy is mathematically formalized as

$$EE=\frac{{𝓗}_{e}}{{𝒩}_{𝒞}}=\frac{-\sum_{i=1}^{\left|S_{abs}\right|}\psi_{i}\log\left(\psi_{i}\right)}{\log\left(\left|S_{abs}\right|\right)},$$ (2)

where preferential exit probability is the probability vector that contains |*S*_*abs*_| entries that represents the likelihood that exit signal selects a specific exiting state *j* ∈ *S*_*abs*_ such that Σ_*j*∈*S*_*abs*__ *ψ*_*j*_ = 1.

The numerator of **EE**(𝒞), that is, −$\sum_{i=1}^{\left|S_{abs}\right|}$ *ψ*_*i*_ log(*ψ*_*i*_), measures the degree to which channels of communication between nodes in *S*_*trans*_ and *S*_*abs*_ are preferred for a fixed task/subject. It is noteworthy that **EE** is not influenced by the (cumulative) magnitudes (of functional connectivity values) that connect nodes from within the FN to outside (exiting) nodes. It is only affected by the distribution of such values. In particular, homogeneous distributions display high entropy levels, and uneven distributions favoring certain exiting node(s) display low entropy. To demonstrate this point, an example is provided in the Supporting Information under the Module Exit Entropy section. The normalizer, 𝒩_𝒞_ = log(|*S*_*abs*_|), is the maximum entropy obtained from a module in which all exit nodes have the same absorption rate. Numerically, a high **EE** would denote the homogeneous integration within the rest of the system, whereas a low **EE** would indicate a preferential communication or integration of the module with the rest of the system. In terms of functional brain networks, module exit entropy facilitates the understanding of collective behavior from 𝒞 to other FNs through its outreach channels (edges formed by nodes in 𝒞 and exiting nodes in *G* \ 𝒞. This is because entropy measures the level of uncertainty in communication; hence, lower entropy means higher specificity in communication between the FN with the rest of the cortex. **EE** value ranges are given in Figure 2.

### The Definition of the Mesoscopic Morphospace Ω

The two distinct features of each FN in brain graphs are addressed by a point **u**(𝒞) in Ω ⊂ (0, *M*) × [0, 1] ⊂ ℝ^2^ as follows:

$$u\left(𝒞\right)=\left(TE\left(𝒞\right),EE\left(𝒞\right)\right)\in \Omega ,$$ (3)

where *M* < ∞. For a given subject and task, a functional brain network *G* is obtained with a predefined parcellation that results in *l* induced subgraph 𝒞 ⊂ *G*. We can then obtain *l* points **u**(𝒞) corresponding to *l* FNs in network *G*.

In general, trapping efficiency **TE**(𝒞) is finitely bounded by construction (see more details in the Module Trapping Efficiency section in the Supporting Information). However, a better bound is possible for the HCP dataset used for this study. This is due to two driving factors: connectome sparsity and edge weights (Avena-Koenigsberger, Goñi, Solé, & Sporns, 2015). We address the upper bound for **TE** as max(**TE**(𝒞)) = *M* = 1. In terms of **EE**(𝒞), its numerical range **EE**(𝒞) ∈ (0, 1]. Hence, Ω ⊂ (0, 1) × [0, 1] for this dataset.

## THE NETWORK CONFIGURAL BREADTH FORMALISM

Studying the manifold topology defined in this 2D mesoscopic morphospace theoretically requires an infinite amount of points. In finite domain with discrete sampling of the morphospace, polytope theory, a mathematical branch that studies object geometry, allows us to create a reasonable scaffold presentation with well-defined properties to formally define and quantify configural components of the functional networks.

Polytope theory is a branch of mathematics that studies the geometry of shapes in a *d*-dimensional Euclidean space, ℝ^*d*^. Given a set of points in this space, *W* = {**x**_1_, **x**_2_, …, **x**_|*W*|_}, a convex hull formed by *W* is represented by

$$\text{Conv}\left(W\right)=\left\{\sum_{j=1}^{\left|W\right|}\alpha_{j}x_{j}|\sum_{j=1}^{\left|W\right|}\alpha_{j}=1,\alpha_{j}\geq 0\right\}.$$

One can compute the notion of volume of the convex hull enclosed by **Conv**(*W*), denoted as *Vol*(**Conv**(*W*)). Given that the morphospace is 2D, the manifold dimension can be from 0 up to 2. In the Supporting Information under the Polytope Theory section, further details on volume computation are defined.

The functional network configural breadth, for the *i*th subject, is compartmentalized into two components:

■  FN (task) reconfiguration and
■  FN rest-to-[task-positive] preconfiguration.

We then propose a mathematical relation between network configural breadth with FN reconfiguration and preconfiguration as follows:

$${𝓕}_{i}=f\left({𝓡}_{i}^{FN},{𝒫}_{i}^{FN}\right),$$ (4)

where 𝓕_*i*_ represents configural breadth for subject *i*th. Here, we provide directly the measures that quantify (functional) reconfiguration and preconfiguration of FNs for *i*th subject’s configural breadth. Tasks are assigned the same level of importance, and hence, no task is weighted more than others.

### Functional Reconfiguration

**Definition 1**. *Functional reconfiguration in this work is represented by a two-dimensional spatial volume derived from given FN’s **EE** and **TE** coordinate values across different cognitive tasks. As such, it represents an example of “cognitive space” (Varona & Rabinovich, 2016; Varoquaux et al., 2018) within a functional domain that spans a variety of network states under various task-evoked conditions. We quantify this as*

$${𝓡}_{i}^{FN}=Vol\left(\text{Conv}\left(W_{i}^{FN}\right)\right),$$ (5)

*where* $W_{i}^{FN}$ *represents the set containing all investigated task coordinates of subject i’s FN;* **Vol**(*Conv*($W_{i}^{FN}$)) *is the convex hull volume induced by points in* $W_{i}^{FN}$*.*

**Figure 3.**
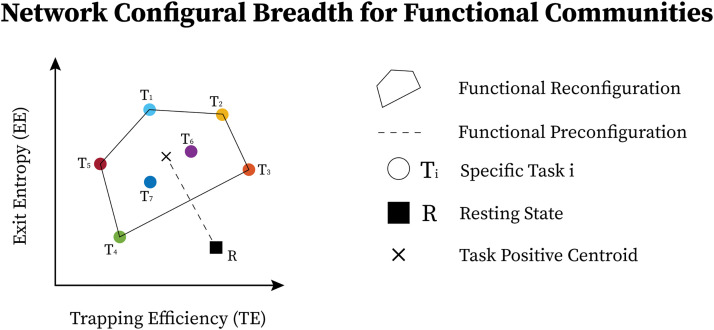
Functional network configural breadth is geometrically represented using two predefined morphospace measures. Specifically, for mesoscopic structures such as communities in functional brain networks, the first measure is trapping efficiency (**TE**) while the second is exit entropy (**EE**). In this case, tasks T1 to T5 belong to the convex hull (e.g., Pareto front; further details are available in the Supporting Information under the Polytope Theory section), while T6 and T7 are in the interior enclosed by the convex hull.

For a given subject *i*th’s FN, note that **Conv**($W_{i}^{FN}$ represents the broad span (breadth) of task configurations for a given functional community. Subsequently, ${𝓡}_{i}^{FN}$ represents the amount of breadth as measured by the volume of **Conv**(*W*). Functional reconfiguration for a given subject’s FN, denoted as ${𝓡}_{i}^{FN}$, is geometrically depicted in Figure 3.

### Functional Preconfiguration

**Definition 2**. *Functional preconfiguration reflects the topologically distributed equipotentiality that is theoretically designed to enable an efficient switch from a resting-state configuration to a task-positive state (Schultz & Cole, 2016), and is quantified as follows:*

$${𝒫}_{i}^{FN}={\left\|{\text{Rest}}_{i}^{FN}-\eta_{W_{i}^{FN}}\right\|}_{2},$$ (6)

*where $\eta_{W_{i}^{FN}}$ is the geometrical centroid of* $W_{i}^{FN}$*;* ${𝒫}_{i}^{FN}$ *measures the distance between rest to task general position (represented by $\eta_{W_{i}^{FN}}$). It is defined with the selected metric space, in this case it is the 2 norm in Euclidean space.*

Note that functional preconfiguration can be viewed as *Vol*(**Conv**(*W*)) where the convex hull is defined solely by two points: FN’s rest and FN’s geometrical centroid of task convex hull, that is, *W* = {$R_{i}^{FN}$, $\eta_{W_{i}^{FN}}$}. In such regards, the notion of *Vol*(**Conv**(*W*)) is also suitable to describe the configural breadth between rest and task-positive location. Functional preconfiguration is geometrically depicted in Figure 3.

## RESULTS

The mesoscopic morphospace formalized in the Mesoscopic Morphospace of Functional Configurations section is used to assess network configural breadth in terms of functional preconfiguration and reconfiguration for the 100 unrelated subjects of the HCP 900-subject data release (Van Essen et al., 2013; Van Essen et al., 2012). This dataset includes (test and retest) sessions for resting state and seven fMRI tasks: gambling (GAM), relational (REL), social (SOC), working memory (WM), language processing (LANG), emotion (EMOT), and motor (MOT). Whole-brain functional connectomes estimated from this fMRI dataset include 360 cortical brain regions (Glasser et al., 2016) and 14 subcortical regions. The functional communities evaluated in the morphospace include seven cortical resting-state FNs from Yeo et al. (2011); visual (VIS), somatomotor (SM), dorsal attention (DA), ventral attention (VA), frontoparietal (FP), limbic (LIM), default mode (DMN), and one composed of subcortical regions (SUBC). Additional details about the dataset are available in the Supporting Information, HCP Dataset and HCP Functional Data sections.

### Task and Subject Sensitivity

#### Within- and between-subject task sensitivity.

**Figure 4.**
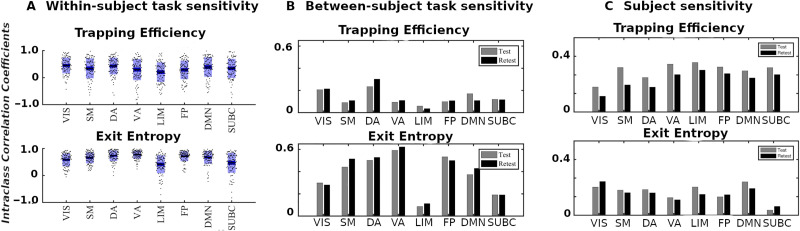
Morphospace measures and their task and subject sensitivity measured by intraclass correlation coefficients for each functional network. (**A**) Within-subject task sensitivity of module trapping efficiency (**TE**) and exit entropy (**EE**) for each FN per subject. (**B**) Between-subject task sensitivity of **TE** (top) and **EE** (bottom). (**C**) Subject-sensitivity ICC of **TE** (top) and **EE** (bottom).

We first evaluate the capacity of module trapping efficiency and exit entropy to differentiate between tasks **within** subject (Figure 4A). For both test and retest sessions of each subject, we compute the **TE** and **EE** metrics for each FN. We compute these values for all eight fMRI conditions. We compute the intraclass correlation coefficient (ICC), with test and retest (per subject) being the repeated measurements and task being the class variable (**TE** in Figure 4A, top and **EE** in Figure 4A, bottom, respectively, where each ICC is computed using a 2 [test, retest] by 7 [tasks] design, and the ICC reflects task within-subject sensitivity). For most subjects, ICC values in all FNs are high and positive values. **EE** displays a higher within-subject task sensitivity than **TE**. Specifically, **TE** in VIS, DA, and DMN most distinguished between the cognitive tasks, whereas **EE** in VA and FP was best at distinguishing the within-subject task-based configural changes. The ICC values for both coordinates were the lowest for LIM.

We then evaluate the degree to which morphospace metrics capture cohort-level configural changes. To test this, for each morphospace metric (**TE** or **EE**), we compute ICC of each FN with subjects as the repeated measures and task as the class variable (Figure 4B). We performed the evaluation separately for test and retest sessions as denoted by gray and dark bars, respectively, for **TE** (Figure 4B, top) and **EE** (Figure 4B, bottom). **EE** captures cohort-level task-based signatures as ICC values are consistently higher than those of **TE**. Interestingly, LIM has the lowest cohort-level task-based sensitivity for both morphospace metrics.

#### Subject sensitivity across tasks.

Here, we compute ICC considering the tasks (fMRI conditions) the repeated measurements and considering subjects the class variable (Figure 4C). It is noteworthy that **TE** is superior in uncovering subject fingerprints, compared with **EE**, for the majority of FNs. This is complementary to **EE** being more task-sensitive.

#### **TE** and **EE** are disjoint features.

Results in the Task and Subject Sensitivity section suggest that **TE** and **EE** have the differentiating capacity to highlight nonoverlapping characteristics of objects under consideration, that is, task- and subject-based FNs. First of all, for within-subject task differentiation (Figure 4A), FNs with high ICC values in one measure do not necessarily show a similar tendency in the other. For instance, VA has the third lowest mean **TE** value in characterizing within-subject task differentiation but it has the highest mean **EE** score. Similarly, FP has the second lowest average **TE** score and the third highest **EE** score, indicating that each of the two measures captures unique aspects of a given FN. Second, evidence of disjoint features is shown through the ICC results in cohort-level task-sensitivity (Figure 4B) and subject-sensitivity (Figure 4C) configural changes. Indeed, **TE** is superior in detecting subject fingerprints, while **EE** is better in unraveling task fingerprints. The idea is that, for a given studied object (i.e., task-based FNs), configurations are shown to “stretch” in exclusive/disjoint directions (subject-sensitive trapping efficiency and task-sensitive exit entropy).

### Quantifying Network Configural Breadth on Functional Networks

The mesoscopic morphospace allows the quantification of network configural breadth. For a given functional community, we compute functional reconfiguration (degree of configurations across tasks) and preconfiguration (distance from rest to task-positive state), using Formulas 5 and 6, respectively.

#### Group-average results.

**Figure 5.**
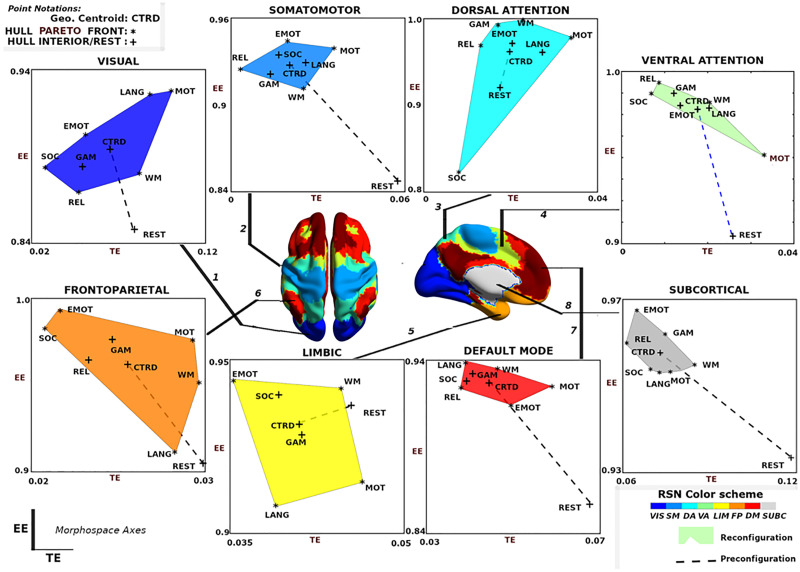
Visualization of network configural breadth. Functional reconfiguration and preconfiguration for all FNs are represented using group average of individual subjects’ coordinates. Task coordinates in this space are represented by either an asterisk (*) or a plus (+) symbol. The asterisk symbol is used for those tasks that are part of the Pareto front of the convex hull; the plus symbol represents either the resting state or task that belongs to the interior of the convex hull. Note that x- and y-axis are purposely not scaled in the same range so that the full range of values for all tasks, task-centroid, and rest can be more easily visualized.

**Figure 6.**
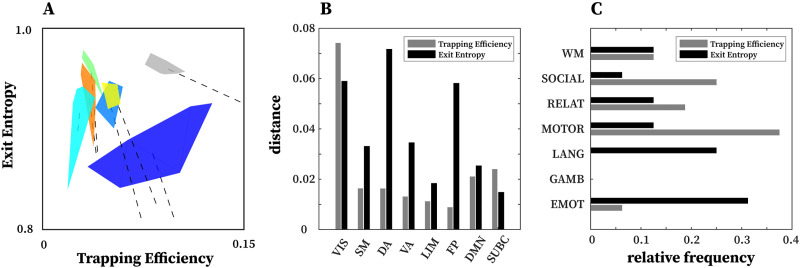
Network configural breadth insights on functional networks and tasks. (**A**) An illustration of network configural breadth for all functional communities. Polytope colors are analogous to the scheme shown in Figure 5. For each functional community, the dashed line represents the amount of functional preconfiguration, whereas the polytope volume represents the amount of functional reconfiguration. (**B**) Maximal distance is computed using the maximum pairwise distance between two tasks for a given functional network. (**C**) Relative frequency with which a task appears in the maximal distance normalized by 16 (8 FNs and 2 tasks per FN).

The group-average behavior of functional communities is shown in Figure 5. Functional reconfiguration of FNs are shown as filled convex hulls, whereas preconfiguration of FNs are shown as dashed lines from rest to the corresponding task hull geometric centroid. To facilitate comparing network configural breadth across all functional networks, these same convex hulls are shown in Figure 6A with the same x- and y-axis values. VIS network polytope, representing group-average behavior, is lower in **EE** relative to other FNs.

With the exception of VIS and SUBC, all other FNs cluster in a similar, high **EE** / low **TE** area of the morphospace (Figure 6A). It should be noted that different tasks and subject populations (e.g., older or clinical groups) might cluster FNs differently. We also note that the subcortical polytope is relatively high in exit entropy. However, the subcortical parcellation might not optimally reflect the functional and/or structural makeup of various subcortical regions (e.g., role of the basal ganglia in the motor system), so these results should be interpreted cautiously.

One observation drawn from such a presentation is that the morphospace framework reconfirms, quantitatively, that functional dichotomy of the brain between task-positive and rest state (Fox et al., 2005). Specifically, the default mode network acts more as a segregated module with high level of integration specificity at rest - as seen in the lower right regime with high **TE**, low **EE** values - as opposed to under task-evoked conditions - as seen in the top left corner with low **TE**, high **EE** values (Figure 5, default mode; Fox et al., 2005; Greicius, Krasnow, Reiss, & Menon, 2003).

Another observation is that in terms of segregation level measured by **TE**, the lower bound of subcortical convex hull is, approximately, the upper bound of other FNs, with the exception of the visual network. Figures 7.1A and 7.2A also summarize functional reconfiguration and preconfiguration, respectively, for test and retest fMRI sessions in all subjects and FNs. Here, the VIS system displays the largest functional reconfiguration (see Figure 7.1A). From Figure 7.2A, functional preconfigurations display a more comparable magnitude among all FNs.

**Figure 7.**
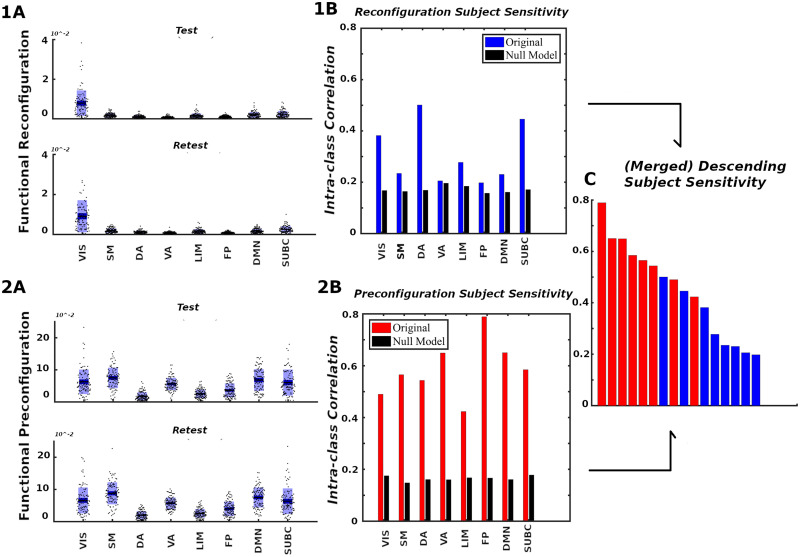
Network configural breadth, subject specificity analysis. Panels **1** and **2** show functional reconfiguration and preconfiguration, respectively, from both magnitude and subject-sensitivity viewpoints. For each functional network, the (**A**) panels report subject’s preconfiguration and reconfiguration values whereas the (**B**) panels quantify subject sensitivity. Reconfiguration and preconfiguration measures are displayed in blue and red, respectively. Panel (**C**) merges all 16 configural breadth terms in descending order of subject sensitivity.

Further evidence of disjoint feature is also displayed in Figure 6B and 6C. In Figure 6B, maximal distance is computed using pairwise distances for two given tasks for a specific FN. The result shows that for a given FN, the two measures complement each other and in many cases, stretch the cognitive space in one direction or the other. For instance, in the case of DA and FP, the maximal distance in **EE** is very high but low for **TE**, whereas in VIS and SUBC, **TE** maximal distance is higher than that of **EE**. Furthermore, in Figure 6C, only specific tasks (e.g., motor and emotion) push the cognitive space in a particular direction (which is captured by maximal distance computation). Evidence of disjoint features is also illustrated by the relative frequency of motor and emotion tasks for which **TE** and **EE** are complementary.

#### Subject specificity of pre- and reconfiguration of functional networks.

The formulation of network configural breadth (in terms of preconfiguration and reconfiguration) enables us to assess these properties at the subject level.

In Figure 7.1B and 7.2B, we use ICC to analyze the ability of morphospace measures (in the form of reconfiguration, panels Figure 7.1, and preconfiguration, panels Figure 7.2) to reflect subject identity within each FN. For all FNs from Yeo et al. (2011), the ICCs suggest that subjects can be differentiated from each other when contrasted against a corresponding null model (for details, see the Supporting Information, Subject Sensitivity section). We see that subject-sensitivity scores of all eight FNs for both pre- and reconfigurations are higher than their corresponding null models. Finally, for a fixed FN, functional preconfigurations dominated the subject sensitivity ranking, as illustrated by Figure 7C. Furthermore, FP, DMN, and VA preconfigurations are among the FNs with the highest subject fingerprints in overall subject-sensitivity ranking.

### Network Configural Breadth and Behavior

Network configural breadth, compartmentalized into FN reconfiguration 𝓡^*FN*^ and preconfiguration 𝒫^*FN*^, shows a high level of subject sensitivity. This allows us to assume that 𝓕_*i*_ is associated with an individual’s behavioral measures (denoted as ⋗_*i*_ for subject *i*th). Several studies reported that FP and DMN networks are associated with memory and intelligence (Gray, Chabris, & Braver, 2003; Schultz & Cole, 2016; Tschentscher, Mitchell, & Duncan, 2017). Therefore, we evaluated whether the outlined framework reflects four widely studied cognitive/behavioral measures, related to memory and intelligence: episodic memory, verbal episodic memory (verb. epi. mem.), fluid intelligence *gF*, and general intelligence *g*. While fluid intelligence reflects subject capacity to solve novel problems, general intelligence, *g*, reflects not only fluid intelligence, *gF*, traits but also crystallized (i.e., acquired) knowledge (Cattell, 1963, and typically denoted as *gC*). The early notion of general intelligence is conceptualized by Spearman’s positive manifold (Spearman, 1904) that cannot be fully described using a single task. Quantification of *g* can be accomplished using subspace extraction techniques such as explanatory factor analysis (Dubois, Galdi, Paul, & Adolphs, 2018) or principal component analysis (PCA; Schultz & Cole, 2016). In this work, we quantified *g* using the PCA approach described in Schultz and Cole (2016). Mathematically, we propose the following composite relationship:

$${⋗}_{i}=ϒ\left({𝓡}_{i}^{FN},{𝒫}_{i}^{FN}\right).$$ (7)

**Figure 8.**
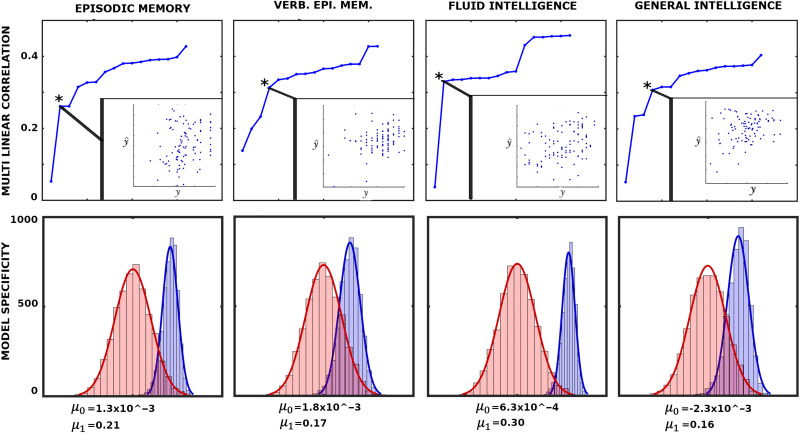
Associations between network configural breadth and behavior. The x-axis represents functional network preconfiguration and reconfiguration terms, that is, ${𝒫}_{i}^{FN}$ and ${𝓡}_{i}^{FN}$, ordered in decreasing subject fingerprints (as shown in Figure 7C). The top panels illustrate iterative multilinear regression model (MLM), while the bottom panels show model specificity (MS) for corresponding behavioral measures. Asterisk represents the optimal MLM with lowest *p* value. Further details are available in the Supporting Information, Behavioral Measure Analysis section.

Having established a plausible connection between behavioral measures and 𝒫^*FN*^, 𝓡^*FN*^, Equation 7 can be viewed as a multilinear model (MLM) using FN preconfiguration and reconfiguration as independent variables (or predictors). The MLM is constructed iteratively, starting with the descriptor with the highest individual fingerprints in Figure 7C. In each iteration, the subsequently ranked descriptor (according to Figure 7C) is appended to the existing ones. The best MLM (denoted with an asterisk in Figure 8), which determines the number of linear descriptors included the model, is selected based on the model *p* value.

To test the level of specificity in the model, we performed 2,000 simulations of *k*-fold cross validation where *k* = 5 between the selected MLM and the corresponding behavioral measure. Specifically, for each cross validation (per simulation), we obtain a correlation between the 20 left-out values (y) with the predicted values (*ŷ*). Hence, in each simulation we obtained five correlations and their mean value. It can be shown that those means follow a normal distribution (details shown in the Supporting Information). Lastly, to provide the level of specificity of linear descriptors, we present a corresponding null model where the same descriptors are evaluated to predict random vectors of appropriate size. To test our model and its ability to predict the behavioral measures, we rely completely on network configural breadth predictors ranked in descending order of subject specificity.

The top panels in Figure 8 show that as more linear descriptors (FN’s functional pre- and reconfigurations) are added to iterative MLMs, variance associating with behavioral/cognitive performance measures decreases with linear descriptors that bear less subject sensitivity. This result highlights the importance of appending linear predictors in descending order with respect to the subject sensitivity. Specifically, as individual specificity reduces from left to right (Figure 7C), the differential correlations, that is, the difference between two consecutive correlation values, decreases.

## DISCUSSION

In this work, we fill an existing gap in the field of network neuroscience by proposing a mathematical framework that captures the extent to which subject-level functional networks, as estimated by fMRI, reconfigure across diverse mental/emotional states. We first propose that brain networks can undergo three different types of (re)configurations: (a) network configural breadth, (b) task-to-task transitional reconfiguration, and (c) within-task reconfiguration. Unlike other existing frameworks (Schultz & Cole, 2016; J. M. Shine et al., 2019; J. M. Shine & Poldrack, 2018), the framework presented here can be applied to all three reconfiguration types. As a first step, we focus on assessing the broadest aspect of reconfiguration, that is, network configural breadth. We postulate, based on previous literature (Cole et al., 2014), that macroscale (whole-brain) and microscale (edge-level) reconfigurations of brain networks are subtle, and hence difficult to disentangle. At the same time, mesoscopic structures in the brain (e.g., functional networks, FNs) reconfigure substantially across different mental/emotional states as elicited by different tasks (Mohr et al., 2016). The framework presented here constitutes the first attempt to formalize such (re)configurations of mesoscopic structures of the brain, and quantify the behavior of a reference set of FNs with changing mental states. We set forth a mathematically well-defined and well-behaved 2D network morphospace using novel mesoscopic metrics of trapping efficiency (**TE**) and exit entropy (**EE**). This morphospace characterizes not only the topology of FNs but also the flow of information within and between FNs. We show that this morphospace is sensitive to FNs, tasks, subjects, and the levels of cognitive performance. We show that both of these measures are highly subject-sensitive for some FNs, while preconfiguration is highly subject-sensitive for all of them. Lastly, we also formalize and quantify the concepts of functional reconfiguration (the extent to which an FN has the capacity to reconfigure across different tasks) and functional preconfiguration (amount of transition from resting-state to a task-positive centroid). We thus construct a formalism that can explore FN changes across different cognitive states in a comprehensive manner and at different levels of granularity.

Ideally, a morphospace framework (Avena-Koenigsberger et al., 2015; Avena-Koenigsberger, Misic, & Sporns, 2018; Corominas-Murtra, Goñi, Solé, & Rodríguez-Caso, 2013; Goñi et al., 2013; McGhee, 1999; Morgan, Achard, Termenon, Bullmore, & Vértes, 2018; Schuetz, Zamboni, Zampieri, Heinemann, & Sauer, 2012; Shoval et al., 2012; Thomas, Shearman, & Stewart, 2000) would have a minimal complexity and, in this particular case, capture distinct features of functional network changes. As discussed in Avena-Koenigsberger et al. (2015), metrics parametrizing a given morphospace should be disjoint. We see that, for any specific FN, high within-subject task sensitivity of **TE** does not necessarily imply a high value in **EE** and vice versa (e.g., VA and FP in Figure 4A). In addition, we see that both **TE** and **EE** offer their unique insights in capturing nonoverlapping features, with **TE** being more subject-sensitive and **EE** more task-sensitive at the cohort level (Figure 4B, 4C). Figure 6B highlights the disjoint nature of the two metrics as well, where we compute maximal distance per FN polytope in the TE and the EE axes separately. Results show that corresponding **TE** and **EE** maximal distances are disjoint and FN dependent. In other words, for a specific FN, the polytope is “stretched” in a particular task direction, where each morphospace measurement (**TE** or **EE**) unravels distinct properties. In Figure 6C, we further see that a subset of tasks dominantly contribute to the maximal distance computation, such as motion, language, and social tasks. Interestingly, we see that motion and language tasks can be considered “orthogonal” tasks with respect to **TE** and **EE**.

Interestingly, the limbic network possesses the lowest ability to distinguish between tasks (Figure 4). Similar behavior has been observed in Amico, Arenas, and Goñi (2019) when using Jensen-Shannon divergence as a distance metric of functional connectivity. In addition, the limbic network seems to work as a “relay” in brain communication (Amico, Abbas, et al., 2019). One potential explanation for this unique behavior is that the limbic network maintains a minimal cognitive load across various tasks, most of which comprises relaying information from one part of the brain to the others; it thus does not reconfigure as much across different mental states.

Brain network configuration is typically studied considering a specific task at multiple spatial and temporal scales (see Bassett et al., 2011; Betzel et al., 2017; Mohr et al., 2016; J. Shine et al., 2018; J. M. Shine et al., 2016; J. M. Shine et al., 2019; J. M. Shine & Poldrack, 2018). Previous investigations have mainly focused on the mechanism of how the brain traverses between high/low cognitive demands (Amico, Arenas, & Goñi, 2019; Avena-Koenigsberger et al., 2018; Bertolero, Yeo, & D’Esposito, 2015; J. M. Shine et al., 2019; Sporns, 2013), or on periods of integration and segregation at rest (J. Shine et al., 2018; J. M. Shine et al., 2019; J. M. Shine & Poldrack, 2018), defined in this paper as within-task reconfigurations. On the other hand, whole-brain configurations have also been investigated across different tasks (one configuration per task) with respect to rest, which led to the concept of general efficiency (Schultz & Cole, 2016). This approach would belong to a wider category that we formally generalize as the network configural breadth. The idea of general efficiency in Schultz and Cole (2016) relied on whole-brain FC correlations between task(s) and rest. While intuitive in quantifying similarity/distance between a single task and rest, quantification across multiple tasks becomes a challenge. Specifically, note that in Schultz and Cole (2016), general efficiency is quantified using the first eigenmode, which explains most of the variance, after measuring the correlation between resting FC and three distinct task FCs. As more and more tasks are included, using the first eigenmode would become less and less representative of the task-related variations present in the data (in this paper summarized as the network configural breadth). The proposed network morphospace overcomes these limitations and can be used to study brain network (re)configurations across any number of tasks. It allows us to study different types of brain network (re)configurations, as mentioned above, using one comprehensive mathematical framework, which also facilitates a meaningful comparison between these seemingly disparate kinds of (re)configurations. Schultz and Cole (2016) proposed that configurations can be compartmentalized into two differentiated concepts: functional reconfiguration and preconfiguration. Note that although the term **reconfiguration** is also used in Schultz and Cole (2016), it is not referring to the action of switching among multiple mental/emotional states, that is, as represented by task-to-task transitional reconfiguration or within-task reconfiguration (as shown in Figure 1B and 1C). Rather, it refers to the overall competence in exploring the total repertoire of task space of each subject given its resting configuration. That is why when we translate the corresponding idea into the mesoscopic morphospace, we call it the network configural breadth. We have also incorporated the two concepts of functional pre- and reconfigurations into a well-defined mathematical space, which solves some of the technical difficulties (as discussed in the Mesoscopic Morphospace of Functional Configurations section) and generalizes these concepts to mesoscopic structures.

Brain network within-task reconfigurations have been almost exclusively qualitatively assessed. For instance, J. M. Shine et al. (2016) show that the whole-brain functional connectome traverses segregated and integrated states as it reconfigures while performing a task. They also found that integrated states are associated with faster, more effective performance. Our formalism of within-task reconfigurations permits assessing such reconfigurations in a quantitative manner. Potentially, such within-task reconfigurations could also be used to assess cognitive fatigue, effort, or learning across time.

Cole et al. (2014) have shown that the resting architecture network modifies itself to fit task requirements through subtle changes in functional edges. Numerically, small changes constituted by functional edges between rest and task-based connectivity might not be statistically significant when looking at edge level. Moreover, we also observe that while such changes might be negligible on a whole-brain global scale, they are more evident when looking at subsystems or functional brain networks, as clearly observed in the VIS network, relative to others. For functional preconfiguration (Figure 5, Figure 6, Figure 7.2A), this effect is observable in all the FNs. In essence, we are postulating that a mesoscopic exploration of changes in brain network configurations with changing mental states is more informative than a macroscopic or microscopic exploration.

A key feature of this morphospace is that, in order to study brain network (re)configuration, an FN is not removed from the overall network for exploration. On the contrary, both metrics that define the morphospace, namely **TE** and **EE**, account for a particular FN’s place embedded within the overall functional brain network, in terms of both topological structure and flow of information. That is why it is important to begin with a reference set of FNs (e.g., RSNs), so as to study how these FNs adapt to changing mental states within the context of the overall network.

Another benefit of a mesoscopic framework is that we can compare individual cognitive traits in each FN, instead of the whole brain (Figure 7.1B, 7.2B). Specifically, after quantifying reconfiguration and preconfiguration for all FNs, we determine whether these quantities incorporate information about individual traits (Figure 7C). We observe different levels of subject fingerprint in different FNs for both re- and preconfiguration measures. This subject fingerprint heterogeneity across different FNs is consistent with previous literature on functional connectome fingerprinting (Amico & Goñi, 2018; Finn et al., 2015). Interestingly, functional preconfiguration (amount of transition from a resting state to a task-positive state) displayed greater subject fingerprint than functional reconfiguration for all FNs. Based on this observation, we argue that to have better subject differentiability, we need to design tasks where the subject transitions from a stable resting state to a task-positive state and/or vice versa (Amico et al., 2020). This could be a significant step forward in precision psychiatry (Fraguas, Díaz-Caneja, Pina-Camacho, Janssen, & Arango, 2016), where we can identify regional brain dysfunction more precisely as a function of the type and degree of cognitive or emotional load.

Subject sensitivity of the proposed network morphospace framework is also supported by significant associations of the frontoparietal and default mode networks with fluid intelligence; see Tables 1 and 2. Specifically, as pointed out by Tschentscher et al. (2017), high fluid intelligence is associated with a greater frontoparietal network activation, which is also consistent with findings from a three-back working memory task (Gray et al., 2003). In the domain of network configural breadth, we observe a higher reconfiguration as represented by a positive frontoparietal functional preconfiguration coefficient (Table 1).

**Table 1.** Multilinear regression models with corresponding standardized *β* coefficients. Dependent variables for each model are episodic memory, verbal episodic memory, fluid intelligence (*gF*), and general intelligence (*g*).

**MLM terms/coefficients**	Constant	𝒫^*FP*^	𝒫^*DMN*^	𝒫^*VA*^	𝒫^*SUBC*^
	*β*_0_	*β*_1_	*β*_2_	*β*_3_	*β*_4_
**Episodic memory**	0.6	2.9	−9.3		
**Verbal episodic memory**	0.5	11.8	−1.1	−8.8	−6.1
*gF*	0.7	5.1	−12		
*g*	0.8	3.9	−5.5	−3.6	−5.7

**Table 2.** Multilinear models with corresponding *p* values. Note that we do not use stepwise linear model which discards descriptors that are not statistically significant. Column entire model shows the significance of the entire model.

**MLM terms/*p* values**	Constant	𝒫^*FP*^	𝒫^*DMN*^	𝒫^*VA*^	𝒫^*SUBC*^	Entire model
	*p*_0_	*p*_1_	*p*_2_	*p*_3_	*p*_4_	
**Episodic memory**	0	0.57	0.01			0.03
**Verbal episodic memory**	0	0.02	0.77	0.17	0.03	0.04
*gF*	0	0.30	9 × 10^−4^			0.004
*g*	0.03	0.44	0.16	0.57	0.05	0.05

This study has several limitations. The framework was tested specifically on the Human Connectome Project dataset and using a single whole-brain parcellation. Alternative parcellations (Schaefer et al., 2018; Tian, Margulies, Breakspear, & Zalesky, 2020), additional fMRI tasks to better sample the cognitive space, and other datasets might offer further insights about the mesoscopic network morphospace (see Avena-Koenigsberger et al., 2015; Corominas-Murtra et al., 2013). In addition, we did not perform a sensitivity analysis on how small fluctuations in functional connectomes affect mapping into the network morphospace. Because of the nature of module trapping efficiency and exit entropy metrics, negative functional couplings were not considered, and hence were set to zero. In future work, other combinations of *L*_1_ and *L*_2_ norms, or even other norm choices, should be evaluated when defining trapping efficiency. This would impact not only the magnitude of the morphospace measure but also the differentiating capacity of configuration across different functional networks.

Future studies should incorporate a sensitivity study of the behavior of this network morphospace with respect to small fluctuations in the input functional connectomes. Further studies could also incorporate structural connectivity information to inform both **TE** and **EE** measures when assessing the morphospace coordinates of functional reconfiguration. Additional exploration of different aspects of this morphospace could provide further insights. For example, location of the polytopes in the morphospace might improve individual fingerprint. An important aspect of the proposed mesoscopic network morphospace is that it allows for an exhaustive and continuous exploration of network reconfigurations, including those that are continuous in time (Douw et al., 2016; J. M. Shine et al., 2019), for example, if the subject performs several tasks within the same scanning session, including extended resting-state periods (such as the fMRI experiment done at Barnes, Bullmore, & Suckling, 2009). This would allow us to fully explore the cognitive space and gain a valuable insight into how different subjects adapt to different levels of cognitive demands. One can also study the trajectory of changing mental states using dynamic functional connectivity (Gonzalez-Castillo et al., 2015), which can easily be mapped to this morphospace for additional insights. Another potential avenue could be the application of this framework to characterize and understand different brain disorders.

In summary, this mesoscopic network morphospace is our first attempt to create a mathematically well-defined framework to explore an individual’s cognitive space at different levels of granularity. It allows us to characterize the structure and dynamics of specific subsystems in the brain. This type of framework can be extremely helpful in characterizing brain dynamics at the individual level, in healthy and pathological populations, which in turn would pave the way for the development of personalized medicine for brain disorders.

## METHODOLOGY

We provide detailed information on materials and methods in the Supporting Information. In short, all necessary mechanics collected from multiple disciplines and general setup for matrix computations are described in main text under the Mesoscopic Morphospace of Functional Configurations section and Supporting Information Preliminaries and Data sections. The dataset consists of high-resolution functional connectivity matrices describing human cerebral cortex and subcortex (see Supporting Information, Data). The construction of morphospace and the formalized notion of configural breadth are described in the Supporting Information, Morphospace Analysis section. Multilinear model and model specificity are described in Supporting Information, Behavioral Measure analysis section.

## ACKNOWLEDGMENTS

Data were provided (in part) by the Human Connectome Project, WU-Minn Consortium (principal investigators: David Van Essen and Kamil Ugurbil; 1U54MH091657) funded by the 16 NIH Institutes and Centers that support the NIH Blueprint for Neuroscience Research; and by the McDonnell Center for Systems Neuroscience at Washington University. JG acknowledges financial support from NIH R01EB022574 and NIH R01MH108467 and the Indiana Clinical and Translational Sciences Institute (Grant Number UL1TR001108) from the National Institutes of Health, National Center for Advancing Translational Sciences, Clinical and Translational Sciences Award. MV and JG acknowledge financial support from Purdue Industrial Engineering Frontier Teams Network Morphospace Award and from Purdue Discovery Park Data Science Award “Fingerprints of the Human Brain: A Data Science Perspective.” We thank Dr. Olaf Sporns and Meenusree Rajapandian for valuable comments.

## SUPPORTING INFORMATION

Supporting information for this article is available at https://doi.org/10.1162/netn_a_00193.

## AUTHOR CONTRIBUTIONS

Duy Anh Duong-Tran: Conceptualization; Formal analysis; Investigation; Methodology; Writing – original draft. Kausar Abbas: Investigation; Writing – original draft. Enrico Amico: Conceptualization; Formal analysis; Methodology; Visualization. Bernat Corominas-Murtra: Conceptualization; Formal analysis; Investigation; Methodology. Mario Dzemidzic: Data curation; Methodology; Writing – original draft. David Kareken: Conceptualization; Supervision; Writing – original draft. Mario Ventresca: Conceptualization; Supervision. Joaquin Goñi: Conceptualization; Data curation; Formal analysis; Funding acquisition; Investigation; Methodology; Project administration; Supervision; Writing – original draft.

## FUNDING INFORMATION

Joaquin Goñi, National Institutes of Health (https://dx.doi.org/10.13039/100000002), Award ID: NIH R01EB022574. Joaquin Goñi, National Institutes of Health (https://dx.doi.org/10.13039/100000002), Award ID: R01MH108467.

## TECHNICAL TERMS

Network configural breadth: Represents, for an FN, a given individual’s repertoire of cognitive and emotional states through functional configurations while performing different tasks. In practice, how well the entire “cognitive space” is sampled depends on the number and nature of the tasks. The functional network configural breadth, for a given subject and a given FN, is compartmentalized into two components: (a) FN (task) reconfiguration and (b) FN rest-to-[task-positive] preconfiguration.
Task-to-task transitional reconfiguration: Represents the specific shift in the network functional configuration of an FN when a subject switches between distinct cognitive/mental tasks. For instance, task transitions and accompanying reconfigurations will occur when a subject transitions from quiet reflection to engage in a spatial problem-solving task, or from a lexical retrieval to a decision-making paradigm.
Within-task reconfiguration: Represents specific network functional configuration changes of an FN that may occur within a single task. This phenomenon has been assessed at the whole-brain level, showing the presence of distinct brain states within a task. For instance, within-task reconfiguration can be tracked by using dynamic (sliding-window) functional connectivity.
Module trapping efficiency (TE): Quantifies the capacity of an FN to act as a segregated module and hence contain (or trap) a signal within its local topology.
Module exit entropy (EE): Quantifies the uncertainty of a signal in taking a specific exiting node while escaping the local topology of an FN.
Functional magnetic resonance imaging (fMRI): A noninvasive imaging modality that estimates brain activity by detecting changes associated with levels of blood oxygenation. The rationale of this technique relies on the fact that there is an association between blood oxygenation and neuronal activation.
Functional reconfiguration: Quantifies the flexibility of an FN as a subject adapts to different cognitive tasks (excluding rest). In this work, it is represented by a two-dimensional spatial volume derived from a given FN’s **EE** and **TE** coordinate values across different cognitive tasks.
Resting-state networks: Spontaneous brain activity is organized into a robust and reproducible (across subjects) set of localized and distributed networks, denoted resting-state networks (RSNs). One of the most common sets of RSNs divides the cortex into seven RSNs: visual (VIS), somatomotor (SM), dorsal attention (DA), ventral attention (VA), limbic (LIM), frontoparietal (FP), and default mode network (DMN). RSNs can be characterized by their functional connectivity in terms of within-network cohesion and between-network integration. RSNs can also be referred to as functional networks (FNs).
Functional connectome/connectivity (FC) matrix: A network representation of the functional coupling between brain regions. Such coupling is usually measured by quantifying the statistical dependencies between time series of brain regions (e.g., pairwise Pearson’s correlation, mutual information) as obtained by functional magnetic resonance imaging (fMRI).
Functional preconfiguration: Reflects, for an FN, the ease of functional transition from a resting-state configuration to a task-positive state. In this work, it is represented using Euclidean distance between **TE** and **EE** coordinates of resting state and geometric centroid of the cognitive tasks.
