# Supplementary material for: A morphospace of functional configuration to assess configural breadth based on brain functional networks
Source: Netw Neurosci. 2021 Aug 30;5(3):666–88. doi: 10.1162/netn_a_00193 (PMC8567831; doi:10.1162/netn_a_00193)
Supplement: Supplementary file 1 [file netn-05-666-s001.pdf]

Duong-Tran, D. A., Kausar, A., Amico, E., Corominas-Murtra, B., Dzemidzic, M., Kareken, D., Ventresca, M., & Goni, J. (2021). Supporting information for "A morphospace of functional configuration to assess configural breadth based on brain functional networks." *Network Neuroscience*. Advance publication. https://doi.org/10.1162/netn_a_00193

Journal: **NETWORK NEUROSCIENCE**

RESEARCH

A morphospace of functional configuration to assess configural breadth based on brain functional networks

Duy Duong-Tran[1 2], Kausar Abbas[1 2], Enrico Amico[1 2 3 4], Bernat Corominas-Murtra[5], Mario Dzemidzic[6], David Kareken[6], Mario Ventresca[1 7]

and Joaquín Goñi[1 2 8 *]

[1]School of Industrial Engineering, Purdue University, West-Lafayette, IN, USA

[2]Purdue Institute for Integrative Neuroscience, Purdue University, West-Lafayette, IN, USA

[3]Institute of Bioengineering/Center for Neuroprosthetics, Ecole Polytechnique Fédérale de Lausanne

[4]Department of Radiology and Medical Informatics, University of Geneva,Switzerland

[5]Department of Zoology, Institute of Biology, Karl-Franzens University Graz, A-8010 Graz, Austria

[6]Department of Neurology, Indiana University School of Medicine, Indianapolis, IN, 46202

[7]Purdue Institute of Inflammation, Immunology and Infectious Disease, Purdue University, West Lafayette, IN, USA

[8]Weldon School of Biomedical Engineering, Purdue University, West Lafayette, IN, USA

[*]Corresponding author: jgonicor@purdue.edu

Keywords: [functional reconfiguration, functional configural breadth, resting state networks, functional connectomes]

SUPPLEMENTAL INFORMATION (SUPPLEMENTAL MATERIALS)

This document purpose is to collaborate on the machinery of the morphospace and other aspects such as the data set and brain atlas used to analyze the data. The aim is to provide further analytic results in conjunction with the ones that are already presented in the main paper.

PRELIMINARIES

In this section, we establish some of the key mathematical notations used throughout the paper. Specifically, scalar is italicized, a. A vector is denoted as bold letter, $\mathbf{a}$ (Default mode is in column fashion). Matrix is notated as capitalized bold letter, $\mathbf{A}$. If $r \in [q]$ where $q \in \mathbb{N}^+$, it means that r accepts integer values from 1 up to, including, q. Given any set S, its cardinality is denoted as $|S|$. Given any two vectors $\mathbf{a}, \mathbf{b} \in \mathbb{R}^n$, $\langle \mathbf{a}, \mathbf{b} \rangle$ denote inner product. In terms of graph theory, a weighted network is denoted as $G(V, E)$ where V and E are sets of vertices and edges in such network, respectively. $G(V, E)$ can be represented by $\mathbf{A}_G = \mathbf{A}(ij) = [w_{ij}]$, in which $w_{ij} \in [0, 1]$ represents functional coupling strength between node i and j. The strength of node $i \in V(G)$ is denoted as k_i, typically stored in the diagonal matrix $\mathbf{K}$ for $\mathbf{K}(ii) = k_i$. A generic matrix $\mathbf{F}$ with entries valued in a continuous interval $[x, y]$, z_1 rows and z_2 columns is denoted as $\mathbf{F} \in [x, y]^{z_1 \times z_2}$. Further, if we want to induce a sub-matrix from the original matrix $\mathbf{F}$ based on a specific set of rows, denoted as set S_{rows}, and columns, denoted as set $S_{columns}$, we use the notation: $\mathbf{F}\big|_{S_{rows}, S_{columns}}$. If the set of rows and columns are matched (both denoted as S_w), then we will ease notation by using $\mathbf{F}\big|_{S_w}$.

DATA

In this section, we provide the details related to the dataset we used to analyze the notion of configural breadth. We also provide information related to the brain atlas.

Brain atlas

The brain atlas used in this work is the based on the cortical parcellation of 360 brain regions as recently proposed by Glasser et al. Glasser et al. (2016). Similarly to reference Amico and Goñi (2018a, 2018b), 14 sub-cortical regions were added, as provided by the HCP release (filename $Atlas\_ROI2.nii.gz$). We accomplish this by converting this file from NIFTI to CIFTI format by using the HCP workbench software
http://www.humanconnectome.org/software/connectomeworkbench.html, with the command -cifti- create-label. This resulted in a brain atlas of 374 brain regions (360 cortical + 14 sub-cortical nodes).

Using Human Connectome Project Dataset, we explore the characteristics of functional networks' configural breadth by utilizing Resting State Networks (FNs), see Yeo et al. (2011), which includes seven

functional networks (FNs): Visual (VIS), SomatoMotor (SM), Dorsal Attention (DA), Ventral Attention (VA), Limbic (LIM), Frontoparietal (FP), Default Mode Network (DMN); Sub-cortical (SUBC) region, as mentioned before, is added into this atlas for completeness. Thus, the parcellation used in this paper comprises of eight (8) FNs.

HCP Dataset

The fMRI dataset used in this paper is available in the Human Connectome Project (HCP) depository (http://www.humanconnectome.org/), with Released Q3. The processed functional connectomes obtained from this data and used for the current study are available from the corresponding author on reasonable request. Please refer to below detailed descriptions on the dataset and data processing.

HCP Functional Data

The fMRI data from the 100 unrelated subjects in the HCP Q3 release were employed in this study Van Essen et al. (2012), Van Essen et al. (2013). Per HCP protocol, all subjects gave written informed consent to the HCP consortium. The two resting-state functional MRI acquisitions (HCP filenames: $rfMRI\_REST_1$ and $rfMRI\_REST_2$) were acquired in separate sessions on two different days, with two distinct scanning patterns (left to right and right to left) in each day, Glasser et al. (2013), Van Essen et al. (2012), and Van Essen et al. (2013) for details. This release includes also data from seven different fMRI tasks: gambling ($tfMRI\_GAMBLING$), relational or reasoning ($tfMRI\_RELATIONAL$), social ($tfMRI\_SOCIAL$), working memory ($tfMRI\_WM$), motor ($tfMRI\_MOTOR$), language ($tfMRI\_LANGUAGE$, including both a story-listening and arithmetic task), and emotion ($tfMRI\_EMOTION$). Per Glasser et al. (2013), Barch et al. (2013), three tasks MRIs are obtained: working memory, motor, and gambling.

The local Institutional Review Board at Washington University in St. Louis approve all the protocol used during the data acquisition process. Please refer to Barch et al. (2013); Glasser et al. (2013); Smith et al. (2013) for further details on the HCP dataset. All tasks and resting functional MRIs are equally weighted importance. In other words, no particular weight is assigned to any specific tasks.

Constructing functional connectomes

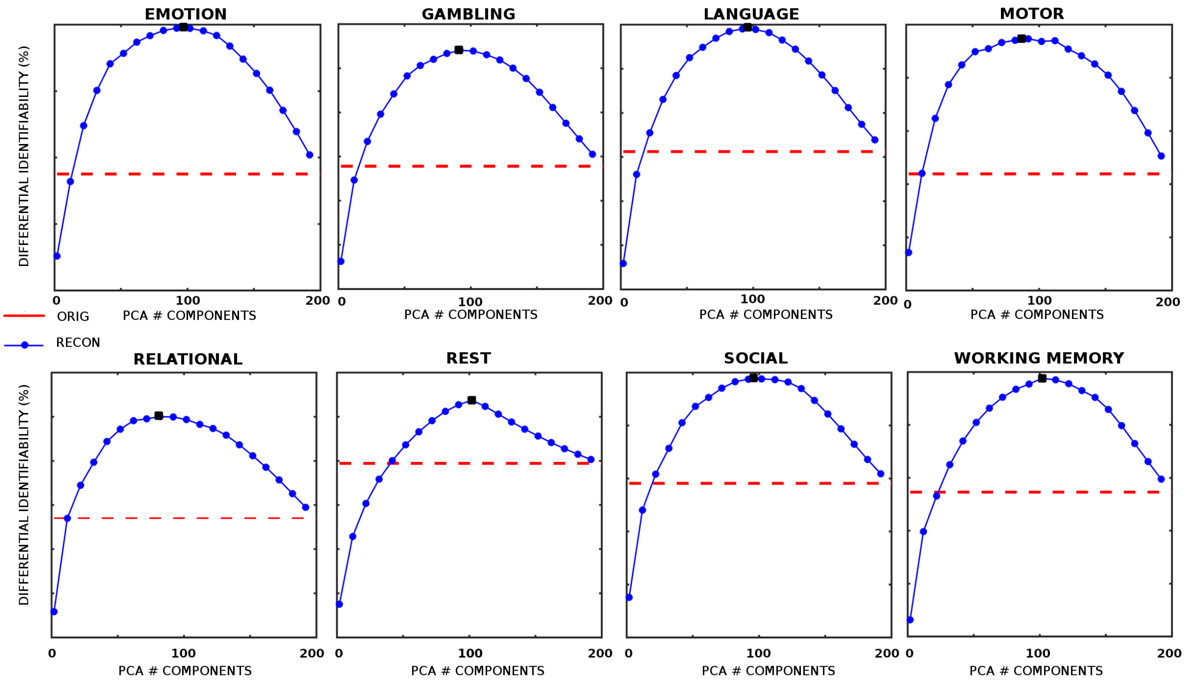

Figure S1: The framework proposed by Amico et al. Amico and Goñi (2018b) is used to maximized individual fingerprints. Each subplot represent rest and seven tasks in HCP dataset. The optimal reconstructed number of orthogonal components is indicated by a black dot.

We used the standard HCP functional pre-processing pipeline, which includes artifact removal, motion correction and registration to standard space, as described in Glasser et al. (2013); Smith et al. (2013) for this dataset. For the resting-state fMRI data, we also added the following steps: global gray matter signal regression; a bandpass first-order Butterworth filter in both directions; z-scores of voxel time courses with outlier eliminations beyond the three standard deviations from first moment Marcus et al. (2011); Power et al. (2014). For task fMRI data, aforementioned steps are applied, with a relaxation for bandpass filter [0.001 Hz, 0.25 Hz]. Starting from each pairs of nodal time courses, Pearson correlation is used to fill out the functional connectomes for all subjects at rest and seven designated tasks. This would yield symmetrical connectivity matrix for all fMRI sections.

Resting State Connectomes: There are two resting scanning sections conducted in two different days. In each day, individual MRIs are obtained independently in the morning and afternoon sections. We

average the resting functional connectome in the first day (which contains morning/afternoon scans) and call it Test. By the same token, we obtain Retest FC for resting condition.

FC's matrix entries: For all considered fMRI images in this paper, we first threshold negative correlations. This is purely technical because one of the morphospace axis is built upon stochastic ground; hence, numerically it is not possible to utilize negative entries. The remaining matrix are, then, squared.

Improve Individual fingerprint To improve identifiability in human functional connectome, we utilize data dimensional reduction technique described in Amico et al. Amico and Goñi (2018b), see **Figure S1**.

MORPHOSPACE ANALYSIS

The concept of a morphospace can be used to analyze many other mathematical objects, including networks. When applied to networks, quantitative traits of global or local network topology are conceptualized through the Cartesian coordinates defined in this abstract space. A brain's subsystem configuration is topologically represented by a point in this multidimensional space. Constructing a morphospace provide us the freedom formally define and track desirable phenotypes of the under-studied objects. In this section, we analyze both measurements in depth. We first introduce the formulation of each axis and then provide further characteristics and/or requirements/assumptions, if any. Any theory that already introduced in the main text will not be re-introduced here.

It is important to be aware that any generic network can be fragmented (disconnected). This is rarely the case for brain functional networks because of how we compute functional couplings, typically using Pearson Correlation Coefficient. Despite of that, *a priori* functional community induced from global thresholded adjacency structure is not guaranteed to be connected. As pointed out in Malliaros and Vazirgiannis (2013) among others, a meaningful cluster should, at minimum, be connected. To respect the global topology of the functional networks G, i.e. connectedness, we applied a small perturbation to edges with zero weight, i.e.

$$a_{ij} = 0 \rightarrow a_{ij} = \epsilon \mid \forall i, j \in \mathcal{C}$$

According to Edelsbrunner and Harer (2010), if one concerns solely on the connectedness property, then topological spaces are similar to graphs. Therefore, the goal is to have the connectedness property carried

from the graph G to all of its induced subgraphs $\mathcal{C}$. To do so, one needs to maintain the topology defined on G, i.e. the collections of open sets $\mathcal{U}$ (which can be thought of as the edge set E defined on G).

The Coordinates of mesoscopic morphospace

In this section, we describe the mesoscopic morphospace Cartesian axes in greater detail. The two coordinates are Module Trapping Efficiency ($\mathbf{TE}$) and Exit Entropy ($\mathbf{EE}$). Thus, for any functional module, $\mathcal{C} \subset G$, we define a point in morphospace Ω to be

$$u(\mathcal{C}) = (\mathbf{TE}(\mathcal{C}), \mathbf{EE}(\mathcal{C}))$$

Module Trapping Efficiency

This is the x-coordinate of $u(\mathcal{C})$. Module trapping efficiency assesses the characteristic of a functional community based on how well it sustains its topology under rich repertoire of task-evoked conditions, relatively to its segregation/integration role, simultaneously. Recall that module Trapping efficiency is formalized as followed:

$$\mathbf{TE}(\mathcal{C}) = \frac{||\tau||_2}{\mathcal{L}_\mathcal{C}}$$

Numerator $\boldsymbol{\tau}$ As claimed in the main text, $\mathbf{TE}$ is finitely bounded. There are several ways to observe this; one approach involves applying hierarchical community detection algorithm Fortunato and Hric (2016) and look for the first time G split into more than one subgraphs. Thus, let i be indices representing communities belong to the first hierarchical layer, then

$$M = \max_k [\mathbf{TE}(S_k)] \mid \forall k \in [l]$$

where l represents the number of communities. Such value is well-defined and finite. An alternative way to see the trivial bound of the measures is as follows: Let us consider the entire network G, we have:

$$\mathbf{TE}(\mathcal{C} \equiv G) = \frac{||\tau||_2}{\mathcal{L}_\mathcal{C}} = \frac{\infty}{0} = \infty$$

because there is no exits if the configurations is the entire network; moreover, there is zero leakages. Hence, any cut into G would have to be strictly less than this upper bound. Note that $\frac{\infty}{0}$ is undefined. However, in such case, we define this quantity to be unbounded which is the notion of infinity.

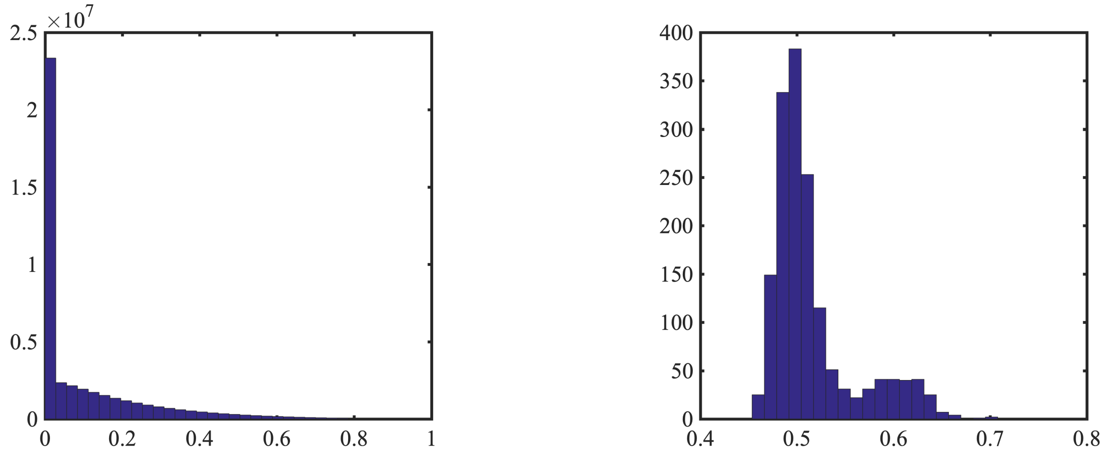

(a) Edge strength, after post-processing steps, histogram of all considered FCs.

(b) FC density, defined to be the number of non-zero functional weighted edges out of $\binom{N}{2}$ possible edges, histogram of all considered FCs.

Figure S2: Mean Density and majority of edge strength falls in the first bin [0,0.025] are the two major factors into the maximum value of **TE**.

In the context of the data set at hand, we can, however provide a better bound then finiteness. We proceed by obtaining the maximum value of **TE** when all subjects and all tasks are under consideration which yields the result

$$\max_{subjects,tasks}(\mathbf{TE}) = 0.5064$$

One can relate this numerical value with two factors: Functional connectome density and edge strengths, see **Fig. S2** for further details.

Normalization Realistically, since larger communities carry more exits which is driven purely from a topological viewpoint, $\mathcal{L}_\mathcal{C}$ is a logical choice to normalize the magnitude of τ.

Additionally, $\mathcal{L}_\mathcal{C}$ is deemed to perform as $||\tau||_2$-damping. Notice that there also exists functional communities with low total exiting strength with large cardinality, theoretically. In such case, these structures are rewarded from the standpoint of $\mathbf{TE}$ as it converges to $\mathbf{TE}(\mathcal{C} \equiv G)$.

Module Exit Entropy

This is the y-coordinate of $u(\mathcal{C})$. Module exit entropy represents communicating preferences of $\mathcal{C}$ with respect to the rest of network G from information theoretical viewpoint. The magnitude of this measure assesses the specificity of integration of a given community in varied environment.

$$\mathbf{EE} = \frac{\mathcal{H}_e}{\mathcal{N}_\mathcal{C}} = -\frac{\langle \psi, log(\psi) \rangle}{log(|S_{abs}|)} = \frac{-\sum_{i=1}^{|S_{abs}|} \psi_i \log(\psi_i)}{\log(|S_{abs}|)}$$

where $\psi^T = [\psi_i]_{i=1}^{i=|S_{abs}|} = \mathbf{1}_{|V_\mathcal{C}|}^T \Psi \left[\mathbf{1}_{|V_\mathcal{C}|}^T \Psi \mathbf{1}_{|S_{abs}|} \right]^{-1}$.

Numerator The numerator of $\mathbf{EE}(\mathcal{C})$, i.e. $-\sum_{i=1}^{|S_{trans}|} \psi_i log(\psi_i)$, measures the extent to which specified channels of communications, under finest scale (i.e. node/edge-level), is established between nodes in $\mathcal{C}$ with nodes that belong to other functional communities in G. Therefore, $-\sum_{i=1}^{|S_{trans}|} \psi_i log(\psi_i)$ captures the properties of the distribution of w_{ij} as a whole, represented by $w_{ij} \forall i \in \mathcal{C}, j \in \mathcal{J}$.

For example, let us say that we have two communities with the same state set $S_{trans} = 1, 2, 3$ and $S_{abs} = a, b, c, d$. In community 1, $w_{ij} = 0.01, \forall i \in S_{trans}, j \in S_{abs}$; and community 2, $w_{ij} = 0.9, \forall i \in S_{trans}, j \in S_{abs}$. Once we compute the entropy, for both cases, we see that they both have no communication preference, hence numerator is one for both cases.

Normalization This is the coordinate where normalization is possible. Note that since entropy is normalized by its maximum value (i.e. $log(|S_{trans}|)$), the number of exits $|S_{trans}|$ impacts is, consequently, neutralized. Thus, one does not need to concern about the cardinality of a community with respect to its number of exits as, in reality, a typically larger community usually carries more exits.

TE, EE behavior across thresholds

In this section, we explore some further characterization of these new metrics used in the morphospace. Specifically, we explore how **TE** and **EE** depend on FC density, across different thresholding values. To do so, we performed the analysis of morphospace measure **TE** and **EE** with different thresholding values 0 to 0.2 at an increment of 0.05. Specifically, for each subject, each task, each FN, and each threshold value, we compute **TE** and **EE**. We then average across subjects, and tasks to obtain **TE** and **EE** for each threshold value.

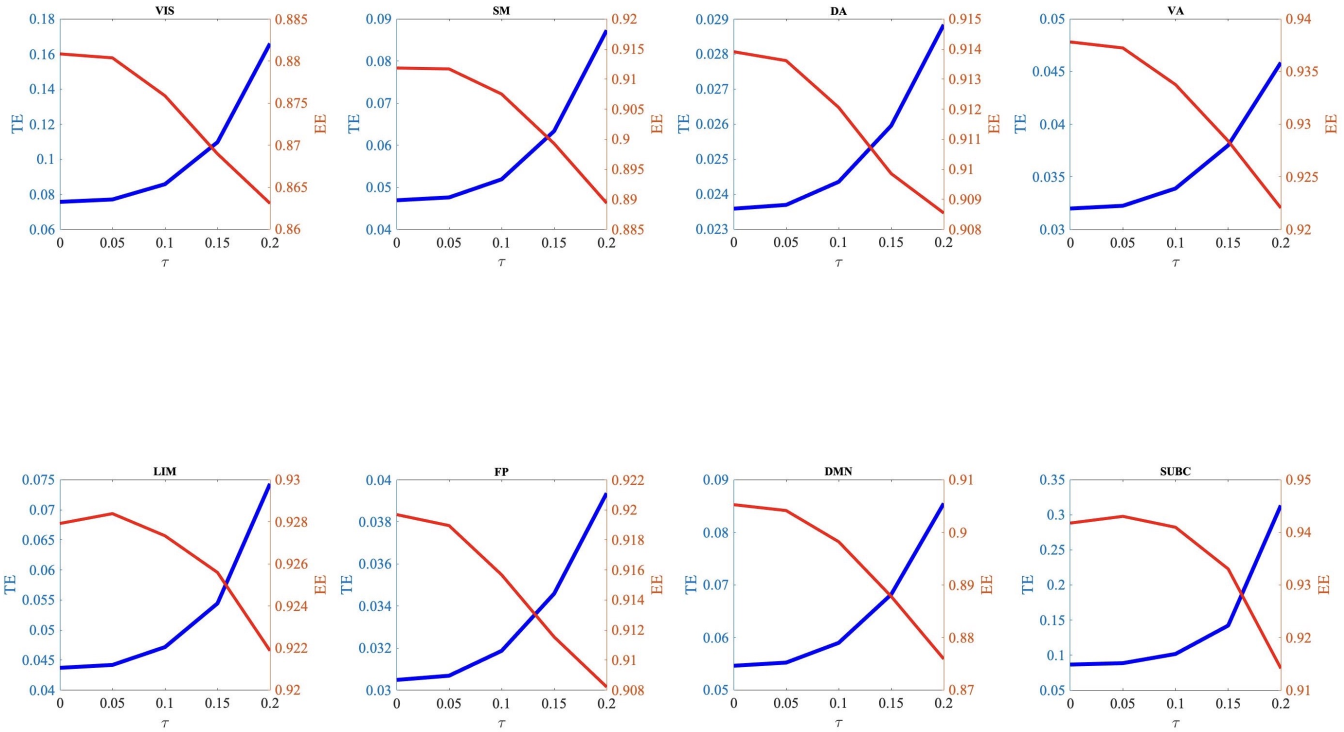

Figure S3: *Morphospace Metric values (**TE** and **EE**) at different thresholds of τ for each functional network within the range* $[0, 0.2]$.

From **Figure S3**, we observe that the values of **TE** and **EE** are stable only for very small magnitudes of the threshold, up to $\tau = 0.075$. As threshold increases, and more functional edges with low-to-intermediate values are removed, the **TE** increases and **EE** decreases. This happens because these edges with low-to-intermediate values, are most likely inter-network connections (i.e. functional edges connecting different FNs) , and their removal makes those networks more segregated, ultimately producing and impact in both **TE** and **EE** measures.

Morphospace Null Model

Randomization of $\boldsymbol{G(V, E)}$ Given a weighted network $A = [w_{ij}]$, we apply randomized algorithm `Xswap`, , see Hanhijärvi, Garriga, and Puolamäki (2009) with number of desired changes that are set to be $[2, 2^3, 2^5..., 2^{19}]$ (with exponent increment of 2) and maximum iterations set at 100 times the

corresponding changes. This algorithm **preserves network basic topological characteristics** such as size, density and degree sequence. As the desired number of changes increases, the difference between the original matrix, denoted as $\mathbf{A}_{orig}$, and the randomized counterpart, denoted as $\mathbf{A}_{rand}$, also increases. The difference between two graphs can be quantified as follows:

$$Diss = \frac{\sum_{i,j=1}^{n} |\mathbf{A}_{rand}(ij) - \mathbf{A}_{orig}(ij)|}{\sum_{i,j=1}^{n} \mathbf{A}_{rand}(ij)}$$

where n is graph's size and $Diss \in [0, 1]$. It is important to note that the difference between two graphs saturates after a certain number of changes and each graph topology saturates at different values (not necessarily 1).

Hence, getting $Diss$ to arbitrarily close to saturation with the smallest number of changes is genuinely the target for this procedure, see **Fig. S4**-Panel **A** for details. In our case, we pick subject 100307 and run the randomization procedure for all available tasks and rest. We first found that the acceptable $Diss$ occurs at 2^{15} desired changes at resting state. We note that the $Diss$ saturates at 0.6 because the (sub)graphs we are dealing with are very dense and some links will be repeated in force, leading to a non-zero overlap between the links of the graphs in the random ensemble and the original one. We then used the same number of changes for the investigated tasks in this dataset.

Morphospace Null Model The main drive for studying trajectories, through randomization, is because it could provide further evidence of the robustness in design of the metrics. Specifically, if done correctly, measurements should highlight unique characteristics of functional communities (and not the randomized counterpart). As the randomized graph (with topological preserved features) get assigned the same partition into functional networks (e.g. Yeo's parcellation) as the original one, any destruction of such topology, at global scale G, would also be carried over (hence, identified) by the morphospace itself. The result is shown in **Fig. S4**. One common theme emerges is that regardless of which functional network and task, as the dissimilarity increases with the desired number of changes, all functional communities are pushed towards to top left corner. This regime of the morphospace represent random exiting strategy from module $\mathcal{C}$ (high value of $\mathbf{EE}$ - high level of uncertainty in communication preference) and high degree of non-assortative community (low $\mathbf{TE}$ - low level of segregation).

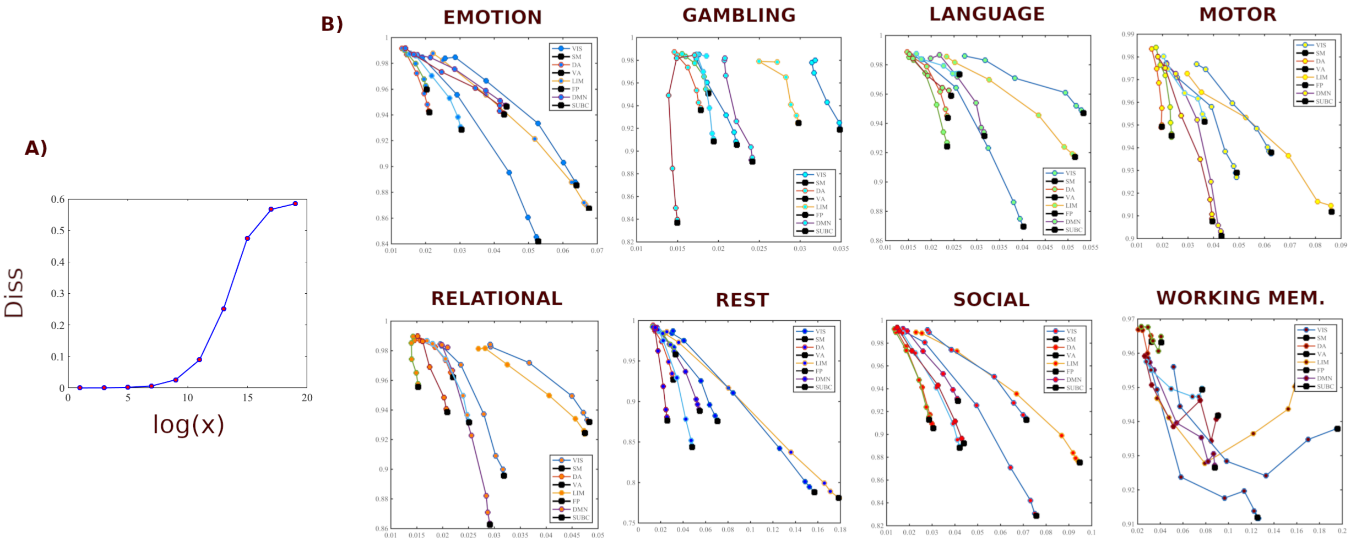

Figure S4: **Morphospace Null Model**: Panel **A)** Subject 100307 with resting connectome using `Xswap` procedure. Panel **B)** represent the application of the selected number of changes to all other tasks. This is an important results to see that functional networks' topology is truly well-defined and highly reproducible across subject domain. Note that black square dot denote functional community **TE** and **EE** with no randomization. Color available online.

NETWORK CONFIGURAL BREADTH

Polytope Theory

Given a set of points $W = \{x_1, x_2, ...x_{|W|}\}$ for which $x_j \in \mathbb{R}^d, \forall j \in [|W|]$, a convex hull formed by such set of points are mathematically represented by

$$\mathbf{Conv}(W) = \left\{ \sum_{j=1}^{|W|} \alpha_j x_j \mid \sum_{j=1}^{|W|} \alpha_j = 1, \alpha_j \geq 0, \forall j \in [|W|] \right\}$$

where d is called the ambient space dimension. Moreover, if $|W| \geq d+1$, we recall that points in W are in general position if no hyperplane, i.e. flat of dimension $d-1$ contains more than d points, Ziegler (2012). Otherwise, i.e. $|W| \leq d$, there exist(s) point(s) that are affinely dependent on other points in W.

Providing that points in W in $\mathbb{R}^d$, the approximated volume induced by the convex hull $\mathbf{Conv}(W)$ can be calculated through the formation of Delaunay Triangulation process Ziegler (2012). The volume of the convex hull is denoted as $\mathbf{Vol}(\mathbf{Conv}(W))$. In $\mathbb{R}^d$, the convex hull dimension can take on the values

1. $h = 0$ which constitutes a point in $\mathbb{R}^d$, $\mathbf{Vol}(\mathbf{Conv}(W)) = 0$
2. $h = 1$ which constitutes a line segment, $\mathbf{Vol}(\mathbf{Conv}(W)) = \sup(d(x_i, x_j)), \forall x_i, x_j \in W$ where $d(x_i, x_j)$ denotes the pre-defined metric distance between two generic points.
3. $h = 2$ and $h \geq 3$ which constitutes the notion of area and volume, respectively.

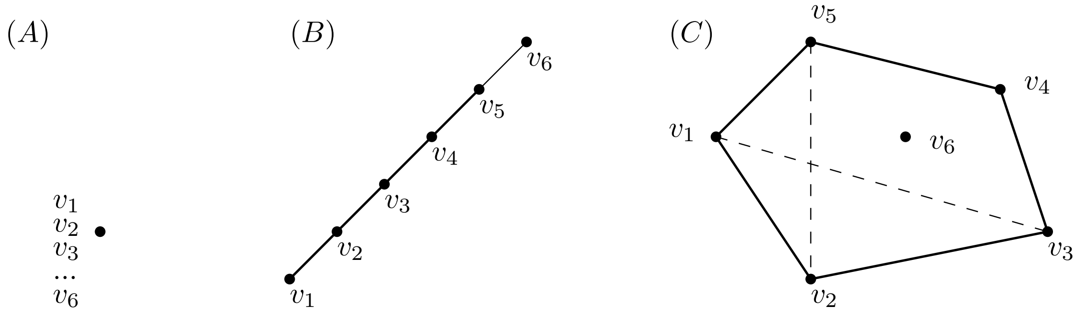

Figure S5: Given that $W = \{v_1, v_2, ..., v_6\}$, we demonstrate three possible scenarios of convex hull formed by W in morphospace Ω. Case *(A),(B),(C)* correspond to the polytope dimension of $h = 0, 1, 2$, respectively. Here we see that $\{v_1, v_6\}$ and $\{v_1, v_2, v_3, v_4, v_5\}$ forms the Pareto front in Case (B) Case (C), respectively. In case (C), v_6 belongs to the interior of the hull. Further, in case (B) and (C), we see that the hull vertices, i.e. points belong to the Pareto front of the hull, are $\{v_1, v_5\}$ for case (B) and $\{v_1, v_2, v_3, v_4, v_5\}$ for case (C). Given the nature of this space, the first two scenarios are statistically rare. In the third scenario, we see that all 5 points constitute the boundary of $\mathbf{conv}(W)$. Further, we see that some type A pairs of points, graphically represented by solid lines, are $(v_1, v_5), (v_2, v_3)$ while some type B pairs, represented by dashed lines, are $(v_2, v_4), (v_3, v_5)$.

For $h \geq 2$, convex hull volume is calculated using `Qhull` package implemented in Matlab, see Page (1995). In general, as pointed out also in Page (1995), computing $\mathcal{V}-$ or $\mathcal{H}-$ polytope metric volume is NP-hard (see also Dyer and Frieze (1988), Khachiyan (1993)) with the availability of efficient approximating algorithms.

Network Configural breadth - A definition

Recall that, in the main text, we define the equivalent notion of configural breadth using functional reconfiguration and preconfiguration for a given FN.

$$\mathcal{F}_i = f(\mathcal{P}_i^{FN}, \mathcal{R}_i^{FN})$$

where $\mathcal{P}_i^{FN}$ and $\mathcal{R}_i^{FN}$ represent functional preconfiguration and reconfiguration, respectively.

Functional Reconfiguration

In the main text, we address that once the points are well-defined to represent tasks per each functional community, we need now the notion that highlights subject capacity to exploring this cognitive space. We provide a deeper analysis of the drive behind the usage of volume of the convex hull.

$$\mathcal{R}_i^{FN} = \mathbf{Vol}(Conv(W_i^{FN}));$$

First of all, since we can only obtain finite number of tasks (hence, points in this space), we see that convex hull notion is logical to represent distinct points (FN tasks) that constitute the Pareto front (hull boundary). To measure the notion of capacity (potential to shift), one needs to measure the notion of spreading given finite number of points in the hull. If we use first order measurements such as distance among two points in the hull, we face the following problems:

- inability to capture the reservoir defined by the interior of the convex hull;
- assumption of linearity between task points

The notion of distance does not cover the space of possibility Avena-Koenigsberger, Goñi, Solé, and Sporns (2015) parameterized by **TE** and **EE**. Hence, second order measurement, i.e. volume (or area in this case), is more appealing.

Functional Preconfiguration

Analogously, once the points are well-defined in this space, in order to effectively measure the notion of functional preconfiguration, we need to highlight the functional readiness, from a cognition standpoint, to switch between resting configuration to a generic task. Here, we first provide the formula proposed in main-text for functional preconfiguration:

$$\mathcal{P}_i^{FN} = ||Rest_i^{FN} - \eta_{W_i^{FN}}||_2$$

where $Rest_i^{FN}$ and $\eta_{W_i^{FN}}$ represent FN coordinate at rest, and geometric centroid considering all FN tasks.

Firstly, the geometric centroid of all FN task coordinates might or might not be cognitively possible, i.e. there might not be a connectome that result in FN task centroid being numerically exact. However, that is not the purpose of using this notion. If the goal is to reflect the degree of functionally readiness between resting and task-engagement, the notion of distance, in this case, is meaningful. Here, complexity of trajectory between rest and task-evoked condition is irrelevant to consider.

Subject Sensitivity

- **Input Matrix**: To quantify subject sensitivity (through configural breadth), for each subject/scan, we obtain one measure. We then concatenate the data into a 100 by 2 matrix and run intra-class correlation (ICC) analysis;
- **Null Model Description**: To test subject sensitivity result robustness, for each functional network's preconfiguration or reconfiguration, we keep one column of ICC input intact (Test) and shuffle the second column (Retest) and measure ICC for each permutation. The same procedure is repeated 10,000 times and the 95%-ile is reported in the main text.

BEHAVIORAL MEASURE ANALYSIS

Iterative Multi-Linear Regression Model (MLM)

Model Description We apply iteratively multi-linear correlation models (MLM) to correlate $\mathcal{F}_i = f(\mathcal{R}_i^{FN}, \mathcal{P}_i^{FN})$ with various behavioral measures, $\gtrdot_i$. We hypothesize that highly subject sensitive predictor, as described in **Fig. 7C** (main text) should be prioritized in MLM model. Iteratively, we start by using only 1 predictor ($\mathcal{P}^{FP}$); in every subsequent step, we append one extra predictor to the existing one(s), again, accordingly per panel **C** of **Fig. 7** (main text). At the end of iterative process, we consequently obtain 16 MLMs.

Optimal MLM - A selection process In order to pick the best MLM (and their corresponding number of linear descriptors in the model), we use the model with smallest p-value among all 16 MLMs.

Model Specificity (MS)

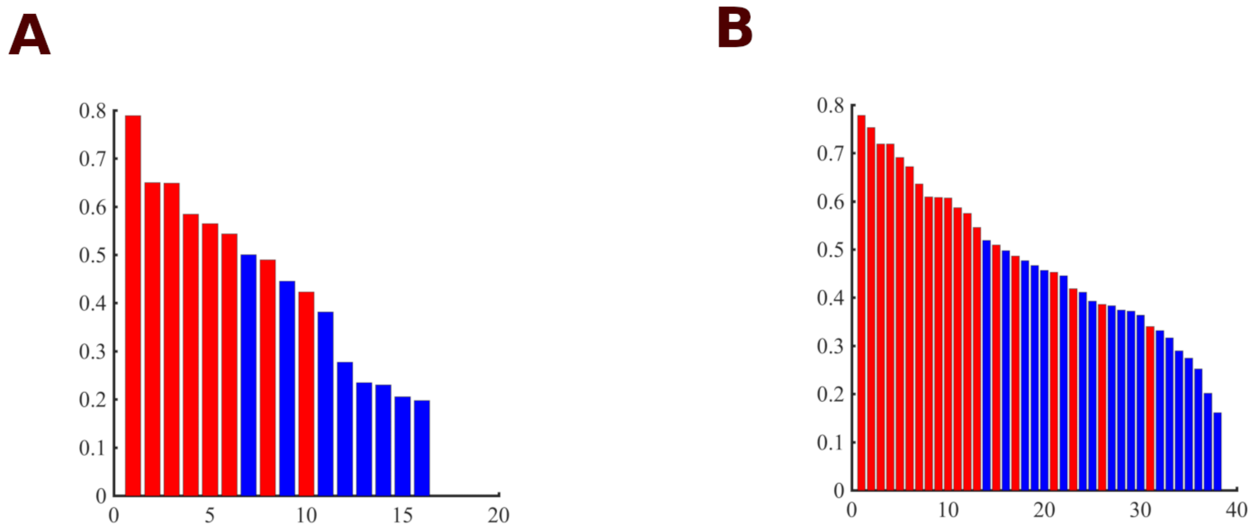

Figure S6: **Network Configural breadth - Subject Specificity Analysis**: FN's pre- (Color: Red) and re- (Color: blue) configuration terms' subject specificity are measured using intra-class correlation. Panel (A) and (B) represent Yeo and colleagues' seven and seventeen FN parcellation Yeo et al. (2011), respectively. In both parcellations, FN preconfigurations, overall, tend to have higher subject fingerprints than reconfiguration.

Constructing the MLM to infer the intrinsic relationship is a necessary but not sufficient if the ultimate goal is to discover if there is a truly robust relationship between network configural breadth and behavioral measures. If there exists such robustness, then there has to be a certain degree of specificity in these models such that only significant correlations are observed when linear predictors are correlated with the true behavioral measures. Specifically, network configural breadth, as mathematically formulated using linear descriptors, must show that it is strongly correlated with a designated measures and not anything else, say a randomized vector.

Model Description We further test the strength of our hypothesis by splitting available data into two subsets: test and validation set. Specifically, we first extract the optimal number of predictors by applying

the procedure described in the main article. We then proceed with the model specificity by creating 2000 simulations; for each simulation - indexed by $j = \{1, 2, 3..., J = 2000\}$ - we first find a randomized order of indices from 1 to 100, denoted as $\vec{d}$, and divide them into five batches (indexed by $i = \{1, 2, ..., I = 5\}$) of 20 subjects. In other words, each batch of 20 randomly picked subjects, indexed by the set Q_i, are used to validate the authenticity of the coefficients proposed by utilizing the remaining 80 unpicked subjects. We see that we recover the permutation of the randomized order vector as follows: $\vec{d} = Q = \cup Q_i$. It is important to note that we use this procedure because it minimizes the chance of picking the same (or highly overlapped) batch of 20 subjects. For each simulation j, in each batch i, the remaining 80 subjects are then used to acquire multi-linear correlation model's parameters, denoted as $\vec{\beta} \in \mathbb{R}^{[*]}$ where $[*]$ denotes the optimal MLM driven by procedure described above (Notice that we use the same notation in the main text under **Fig. 8** as well). These corresponding coefficients are then used to predict the remaining 20 unused data points, indexed by $w \in W_i$, denoted as $\hat{y}$.

$$\hat{y}_w = \vec{\beta}_0 + \left[\left\{\mathcal{P}_w^{FN}, \mathcal{R}_w^{FN}\right\}^{[*]}\right] \vec{\beta}$$

where $\left\{\mathcal{P}_w^{FN}, \mathcal{R}_w^{FN}\right\}^{[*]} \in \mathbb{R}^{+,[*]}$ is the $[*]$-tupled vector representing functional preconfiguration, reconfiguration, obeying the descending order of concatenated subject sensitivity in **Fig. 7C** located in the main text. Next, for each batch, we compute the correlation between actual values, y_w with predicted ones, $\hat{y}_w$ and record the correlating result, denoted as $R_i, \quad \forall i = 1, 2, ..., I = 5$. Consequently, at each simulation, we obtain 5 values of R_i corresponding to 5 batches. Lastly, for each simulation j, the mean and standard deviation of 5 validation models R_i's is obtained

$$R_j = \sum_{i=1}^{I} R_{ij} = \langle R_{:,j} \rangle \quad \& \quad \sigma_j = \sqrt{\frac{\sum_{i=1}^{I} (R_{:,j} - R_j)^2}{I}}$$

Per Central Limit Theorem, the statistic $R_j \mid \forall j = \{1, 2, ..., J = 2000\}$ is normally distributed, i.e. $R_j \sim N(\mu_0, \sigma_0)$. This would create an empirically normal distribution $R_j \sim N(\mu_0, \sigma_0)$ such that

$$\mu_0 = \frac{\sum_j \sum_i R_{ij}}{I \times J} \quad \& \quad \sigma_0 = \sqrt{\frac{\sum_{j=1}^{J} \sigma_j^2}{J}}$$

MS's null model and paired t-test Similarly to the MLMs, we want to test the authenticity of selected models by testing it against artifacts such as random vectors. The same procedure is applied for the

random vector to populate the null model's empirically normal distribution (its means is notated as μ_1): $R_j^{rand} \sim N(\mu_1, \sigma_1)$. Finally, paired t-tests are applied between the two aforementioned distributions, i.e. R_j and R_j^{rand}, to test the capacity of configural breadth predictors towards behavioral measures. Interestingly, given the investigated behavioral measures, all null model empirical distributions have very similar first and second moments, independently on behavioral measures.

REFERENCES

Amico, E., & Goñi, J. (2018a). Mapping hybrid functional-structural connectivity traits in the human connectome. *Network Neuroscience*, 1–17.

Amico, E., & Goñi, J. (2018b). The quest for identifiability in human functional connectomes. *Scientific reports*, *8*(1), 8254.

Avena-Koenigsberger, A., Goñi, J., Solé, R., & Sporns, O. (2015). Network morphospace. *Journal of the Royal Society Interface*, *12*(103), 20140881.

Barch, D. M., Burgess, G. C., Harms, M. P., Petersen, S. E., Schlaggar, B. L., Corbetta, M., … others (2013). Function in the human connectome: task-fmri and individual differences in behavior. *Neuroimage*, *80*, 169–189.

Dyer, M. E., & Frieze, A. M. (1988). On the complexity of computing the volume of a polyhedron. *SIAM Journal on Computing*, *17*(5), 967–974.

Edelsbrunner, H., & Harer, J. (2010). *Computational topology: an introduction*. American Mathematical Soc.

Fortunato, S., & Hric, D. (2016). Community detection in networks: A user guide. *Physics Reports*, *659*, 1–44.

Glasser, M. F., Coalson, T. S., Robinson, E. C., Hacker, C. D., Harwell, J., Yacoub, E., … others (2016). A multi-modal parcellation of human cerebral cortex. *Nature*, *536*(7615), 171–178.

Glasser, M. F., Sotiropoulos, S. N., Wilson, J. A., Coalson, T. S., Fischl, B., Andersson, J. L., … others (2013). The minimal preprocessing pipelines for the human connectome project. *Neuroimage*, *80*, 105–124.

Hanhijärvi, S., Garriga, G. C., & Puolamäki, K. (2009). Randomization techniques for graphs. In *Proceedings of the 2009 siam international conference on data mining* (pp. 780–791).

Khachiyan, L. (1993). Chapter iv. complexity of polytope volume computation. *New trends in discrete and computational geometry*, *10*, 91.

Malliaros, F. D., & Vazirgiannis, M. (2013). Clustering and community detection in directed networks: A survey. *Physics Reports*, *533*(4), 95–142.

Marcus, D., Harwell, J., Olsen, T., Hodge, M., Glasser, M., Prior, F., … Van Essen, D. (2011). Informatics and data mining tools and strategies for the human connectome project. *Frontiers in neuroinformatics*, *5*, 4.

Page, T. G. C. H. (1995). *Qhull the geometry center home page.* Retrieved 1995-05-17, from http://qhull@qhull.org

Power, J. D., Mitra, A., Laumann, T. O., Snyder, A. Z., Schlaggar, B. L., & Petersen, S. E. (2014). Methods to detect, characterize, and remove motion artifact in resting state fmri. *Neuroimage*, *84*, 320–341.

Smith, S. M., Beckmann, C. F., Andersson, J., Auerbach, E. J., Bijsterbosch, J., Douaud, G., … others (2013). Resting-state fmri in the human connectome project. *Neuroimage*, *80*, 144–168.

Van Essen, D. C., Smith, S. M., Barch, D. M., Behrens, T. E., Yacoub, E., Ugurbil, K., … others (2013). The wu-minn human connectome project: an overview. *Neuroimage*, *80*, 62–79.

Van Essen, D. C., Ugurbil, K., Auerbach, E., Barch, D., Behrens, T., Bucholz, R., … others (2012). The human connectome project: a data acquisition perspective. *Neuroimage*, *62*(4), 2222–2231.

Yeo, B. T., Krienen, F. M., Sepulcre, J., Sabuncu, M. R., Lashkari, D., Hollinshead, M., … others (2011). The organization of the human cerebral cortex estimated by intrinsic functional connectivity. *Journal of neurophysiology*, *106*(3), 1125–1165.

Ziegler, G. M. (2012). *Lectures on polytopes* (Vol. 152). Springer Science & Business Media.
